# Synthesis and Evolution of Berberine Derivatives as a New Class of Antiviral Agents against Enterovirus 71 through the MEK/ERK Pathway and Autophagy

**DOI:** 10.3390/molecules23082084

**Published:** 2018-08-20

**Authors:** Yan-Xiang Wang, Lu Yang, Hui-Qiang Wang, Xiao-Qiang Zhao, Ting Liu, Ying-Hong Li, Qing-Xuan Zeng, Yu-Huan Li, Dan-Qing Song

**Affiliations:** Beijing Key Laboratory of Antimicrobial Agents, Institute of Medicinal Biotechnology, Chinese Academy of Medical Sciences and Peking Union Medical College, Beijing 100050, China; wangyanxiang@imb.pumc.edu.cn (Y.-X.W.); yolo2022@163.com (L.Y.); hq_wangimb@163.com (H.-Q.W.); xiaoqiangzhao2017@126.com (X.-Q.Z.); lutyliu@126.com (T.L.); liyinghong@imb.pumc.edu.cn (Y.-H.L.); zqx50810793@163.com (Q.-X.Z.)

**Keywords:** enterovirus 71, berberine, structure-activity relationship, MEK/ERK pathway, autophagy

## Abstract

Taking berberine (BBR) as the lead, 23 new BBR derivatives were synthesized and examined for their antiviral activities against four different genotype enterovirus 71 (EV71) strains with a cytopathic effect (CPE) assay. Structure-activity relationship (SAR) studies indicated that introduction of a suitable substituent at the 9-position might be beneficial for potency. Among them, compound **2d** exhibited most potent activities with IC_50_ values of 7.12–14.8 μM, similar to that of BBR. The effect of **2d** was further confirmed in a dose-dependent manner both in RNA and protein level. The mechanism revealed that **2d** could inhibit the activation of MEK/ERK signaling pathway. Meanwhile, it could suppress the EV71-induced autophagy by activating AKT and inhibiting the phosphorylation of JNK and PI3KIII proteins. We consider BBR derivatives to be a new family of anti-EV71 agents through targeting host components, with an advantage of broad-spectrum anti-EV71 potency.

## 1. Introduction

Enterovirus 71 (EV71), a single-stranded positive-sense RNA virus belonging to the enterovirus genus of the *Picornaviridae* family, is the primary cause of the hand, foot and mouth disease (HFMD), which is widely spread among infants and young children, especially those under 5 years old [1,2]. EV71 was first isolated from patients with central nervous system (CNS) diseases in California in 1969 and HFMD caused by EV71 infections usually is self-limiting, but some EV71-caused HFMD cases have been associated with neurological diseases such as aseptic meningitis, myocarditis and pulmonary edema [3,4,5]. Every year, from April to July, HFMD has a high incidence, which has become a seriously social and public health concern in mainland China. However, up to now there is still no effective drug for the prevention and treatment of HFMD in the clinic. This situation has resulted in a very pressing need for the discovery of novel anti-EV71 drug candidates for the control of infectious diseases arising from EV71 [6,7].

**Figure 1:**
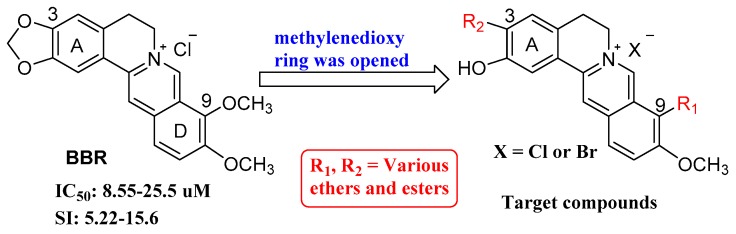
Chemical structure of BBR, and structure modification strategy.

We have been working to find innovative drug candidates from Chinese natural products such as berberine (BBR, Figure 1), which has been widely used in China for decades against diarrhea as a Traditional Chinese Medicine (TCM). We have continuously reported that BBR derivatives possess various pharmacological effects, such as antibacterial [8], anti-Coxsackie virus [9], anti-inflammatory [10], immunotherapeutic [11] and anti-tuberculosis activities [12]. Recently, we have first identified that BBR exerted a moderate activity against EV71 replication with new mechanism of action, mainly through down-regulating EV71-induced autophagy and MEK/ERK signaling pathway [4]. The unique chemical scaffold and biological activity against EV71 of BBR spurred us to further conduct structural modifications and optimization of its kind, so as to explore structure-activity relationship (SAR) against EV71 as well as acquire promising anti-EV71 candidates.

Based on the strategy, a series of new BBR derivatives, including variety esters and ethers on positions 3 and 9 as depicted in Figure 1, were prepared due to the starting material availability and synthetic ease, and evaluated for their anti-EV71 activity taking BBR as the lead. In the present study, we described the synthesis of 23 new BBR derivatives, SAR analysis and primary mechanism of action of the representative compound.

## 2. Results and Discussion

### 2.1. Chemistry

**Scheme 1:**
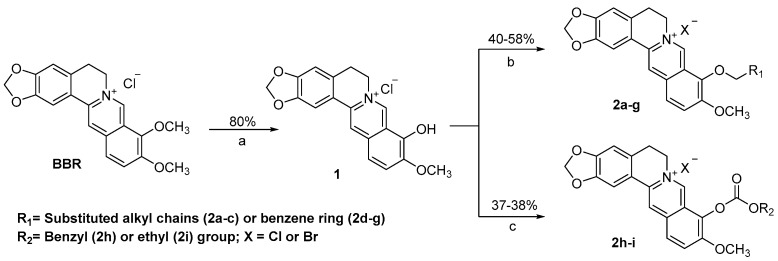
*Reagents and conditions*: (**a**) 195 °C, 30–40 mmHg, 1 h; (**b**) R_1_Br, K_2_CO_3_, DMF, 70 °C; (**c**) R_2_Cl, K_2_CO_3_, CH_3_CN, 70 °C.

**Scheme 2:**
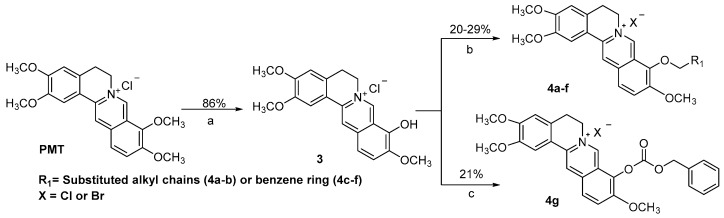
*Reagents and conditions*: (**a**) 195 °C, 30–40 mmHg, 1 h; (**b**) R_1_Br, K_2_CO_3_, DMF, 70 °C; (**c**) Benzyl chloroformate, K_2_CO_3_, CH_3_CN, 70 °C.

**Scheme 3:**
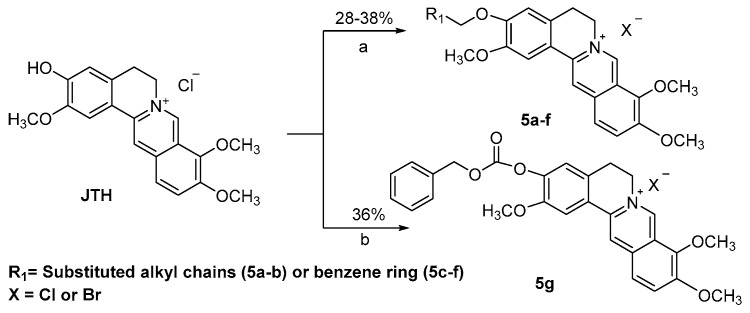
*Reagents and conditions*: (**a**) R_1_Br, K_2_CO_3_, DMF, room temperature; (**b**) Benzyl chloroformate, K_2_CO_3_, CH_3_CN, room temperature.

A total of twenty-three new BBR derivatives were semi-synthesized as displayed in Scheme 1, Scheme 2 and Scheme 3, taking commercially available BBR, palmatine (PMT) or jatrorrhizine (JTH) as the starting material, respectively. All the ^1^H-NMR, ^13^C-NMR, HRMS-ESI spectra data of target compounds can be found in Supplementary Materials. As shown in Scheme 1, the key intermediate **1** was obtained under vacuum from BBR as reported previously [10,11]. Then, BBR ethers **2a**–**g** were obtained by alkylation of compound **1** with corresponding bromides with K_2_CO_3_ as the base with yields of 40–58%. Similarly, BBR esters **2h** and **2i** were prepared through esterification of compound **1** [13] with corresponding chlorides in 37% and 38% yield, respectively.

As described in Scheme 2, intermediate **3** [14] was synthesized after demethylation of PMT on position 9. Compounds **4a**–**f** were prepared via alkylation with K_2_CO_3_ as the base in overall yields of 20–29%, while compound **4g** was created by esterification of compound **3** by 21% yield.

Finally, as depicted in Scheme 3, the ether products **5a**–**f** were directly obtained via alkylation using corresponding bromides at room temperature from starting material JTH. JTH was esterified with benzyl chloroformate under alkaline condition to obtain compound **5g** by 36% yield. All the final products were purified via flash column chromatography using CH_3_OH/CH_2_Cl_2_ as the gradient eluent.

### 2.2. Pharmacological Evaluation

#### 2.2.1. SAR for Anti-EV71 Activity

Thus, all newly synthesized BBR analogues were screened for their anti-EV71 potencies in African green monkey kidney (Vero) cells taking BBR as the positive control. EV71 strains used in the present study include different genotype strains, such as H strain (VR-1432, genotype C2), BrCr strain (VR-1775, genotype A), and JS-52 and SHZH98 (genotype C4). The potency against EV71 strains above mentioned of each tested compound was evaluated by the combination of its IC_50_ and selectivity index (SI) value as the important therapeutic indication. The structure and anti-EV71 activity of each new compound are shown in Table 1.

First, the key intermediates **1**, **3**, and JTH were screened against H strain of EV71, and all of them showed decreased activity compared with that of BBR. SAR analysis was then focused on the influence of substitutions on position 9 of ring D, and seven ether derivatives (**2a**–**g**) and two ester analogues (**2h** and **2i**) were prepared and tested, respectively. As described in the table, among them, ether compounds **2d**–**g** with different substituents on the benzene ring displayed satisfactory potencies with IC_50_ values of 12.4 and 15.8 μM, especially compound **2d** that exhibited lower cytotoxicity and a better SI value of 6.7, similar to that of BBR. Meanwhile, the carboxylic acid form of compound **2d** (**2d**-**a**) exhibited a complete loss on the antivirus potency which indicated that compound **2d** might exert the activity on its original form. Two ester derivatives **2h** and **2i** gave obvious decreased activity, The results hinted that introducing a suitable ether substituent on position 9 might be beneficial for the ability against EV71.

In the second round of SAR study, the methylenedioxy ring was opened and substituent on position 9 of ring D was attached, by which seven analogues **4a**–**g** were created. Only compound **4e** with a 9-*m*-methoxybenzyloxycarbonylmethylenoxy moiety displayed a comparable activity to BBR. Meanwhile, while methylenedioxy ring was opened and the substituent was switched to position 3 of ring A, compounds **5a**–**g** were prepared and screened. Among them, compound **5e** gave ideal activities compared with BBR as well as high cytotoxicity. The inhibitory activity of compounds **5a**–**d**, **5f** and **5g** was partially or completely lost.

Next, the anti-EV71 activities of all the target compounds against JS-52, SHZH98 and BrCr strains were also tested respectively. As shown in Table 1, the screening results were almost consistent with that of H strain. These results indicated that appropriate substituents on position 9 and methylenedioxy on ring A might be beneficial for the antivirus potency. Among them, compound **2d** exhibited most satisfactory potency against all tested genotype EV71 strains as well as high SI values, which indicated that compound **2d** exhibited a broad-spectrum anti-EV71 activity, and was selected as the representative compound for further investigation.

**Figure 2:**
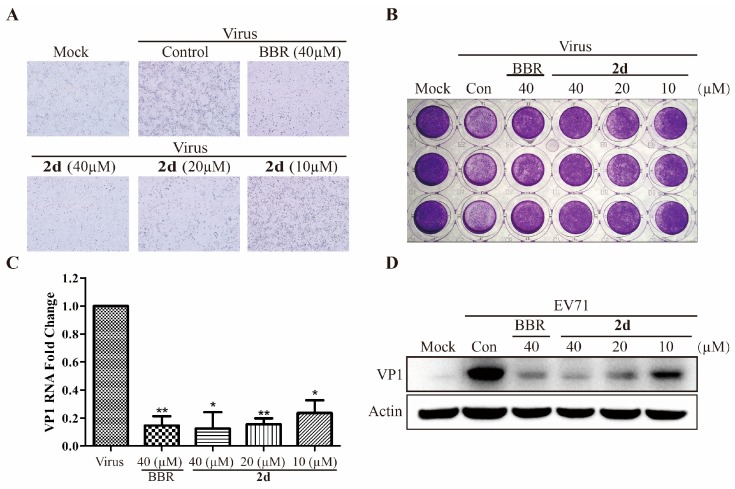
The anti-EV71 effect of compound **2d** in Vero cells with BBR as a positive control. (**A**) **2d** reduced the EV71-induced CPE. Cells were examined using a microscopy (×40); (**B**) **2d** reduced the EV71-induced CPE. Cells were examined using crystal violet staining; (**C**) **2d** reduced the expression of EV71 VP1 RNA by one-step qRT-PCR assay. ** *p* < 0.001 * *p* < 0.05; (**D**) **2d** reduced the expression of EV71 VP1 protein by western blot assay.

Furthermore, microscopy and crystal violet staining was used to further investigation the anti-EV71 effect of **2d** with CPE (cytopathic effect) assay [4]. As shown in Figure 2A,B, the anti-EV71 effect of **2d** was visually demonstrated that it could significantly reduce the EV71-induced CPE at the concentration of 20 μM, better than that of BBR.

#### 2.2.2. Compound **2d** Inhibits EV71 Replication in Both RNA and Protein Level

To further confirm the anti-EV71 activity, the VP1 down-regulatory activity of **2d** was examined in Vero cells infected with H strain of EV71, in comparison with that of BBR. As shown in Figure 2C, compound **2d** significantly reduced VP1 RNA expression in a dose-dependently way, and the activity of **2d** was better than that of BBR in the reverse transcription-quantitative polymerase chain reaction (RT-qPCR) assay. Accordingly, VP1 capsid protein expression was also analyzed to examine its effect on EV71 biological synthesis. As depicted in Figure 2D, compound **2d** could obviously down-regulate the expression of VP1 protein in a dose-dependent manner, and compound **2d** exhibited better antiviral effect than that of BBR at the concentration of 40 μM.

### 2.3. Compound ***2d*** Inhibits the Phosphorylation of MEK/ERK

**Figure 3:**
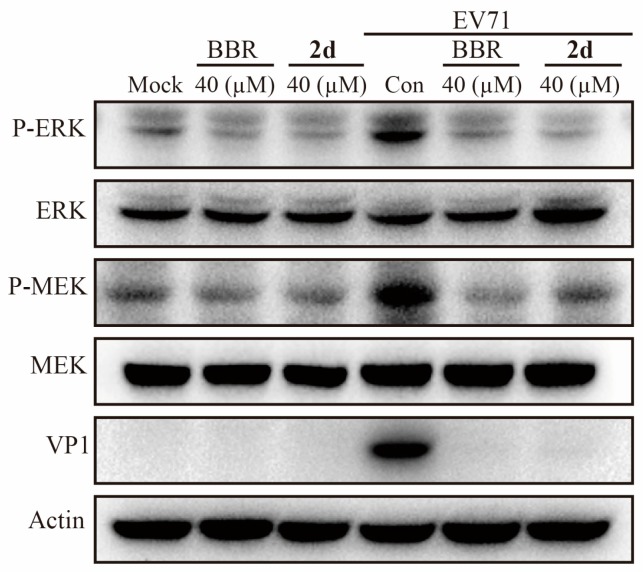
Compound **2d** inhibits the phosphorylation of MEK/ERK with BBR as a control. Vero cells (9 × 10^5^ cells/well) were plated into 6-well culture plates and were mock-infected or infected with EV71 (H, MOI = 0.1) for 1 h. The cells were then treated with compound **2d** for 24 h and were harvested for western blot assay.

The activation of the MEK/ERK signaling pathway has been certified to play an essential role in EV71 life cycle and pathogenesis in various cell types [15,16,17,18,19,20,21,22]. We have first confirmed that BBR could inhibit the phosphorylation of MEK/ERK induced by EV71 infection [4]. To learn whether or not **2d** would still act through MEK/ERK pathway as its parent BBR does, the inhibition experiment of EV71 via this signaling pathway was done with **2d** using BBR as the reference. As shown in Figure 3, compound **2d** could significantly inhibit the phosphorylation of both MEK and ERK induced by EV71 infection, so as to attenuate the activation of MEK/ERK signaling pathway. We can conclude that compound **2d** inhibited the replication of EV71 through down-regulating MEK/ERK signaling pathway.

### 2.4. Compound ***2d*** Inhibits EV71-Induced Autophagy

**Figure 4:**
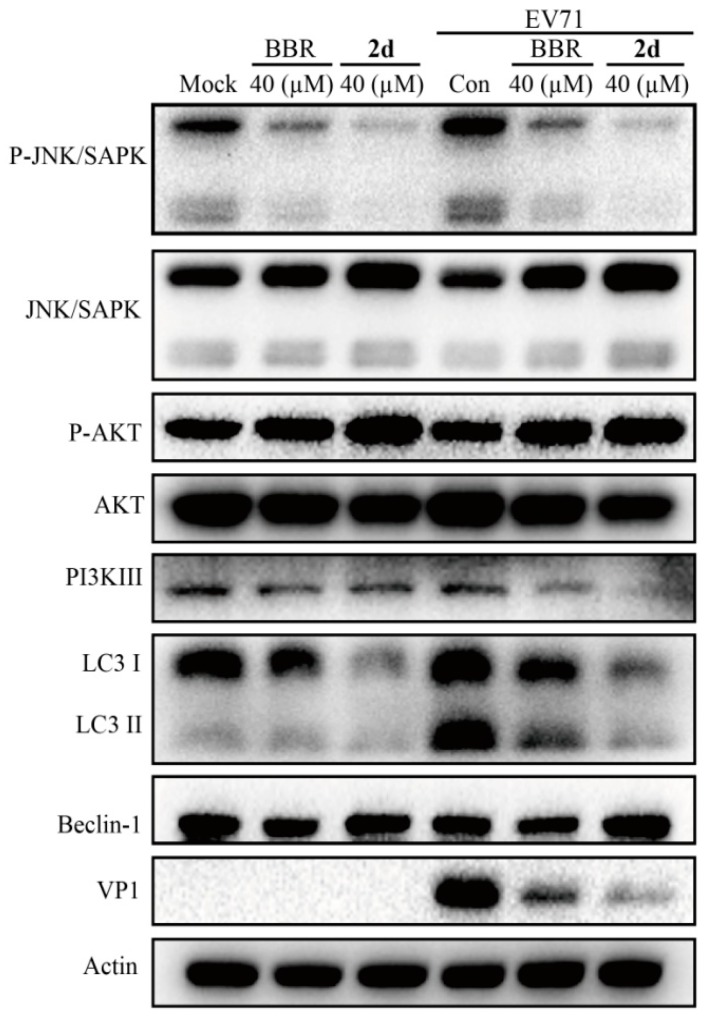
Compound **2d** inhibits EV71-induced autophagy. Vero cells were mock-infected or infected with EV71 (H, MOI = 0.1) for 1 h. The cells were then treated with compound **2d** and BBR for 24 h. The cells were harvested and proteins were examined by western blot.

EV71 infection triggers autophagy, which provides a support for EV71 replication [23]. Both JNK and PI3KIII proteins as upstream activators, play important roles in regulating autophagy, and inhibition of corresponding signaling pathway could down-regulate autophagy [24]. Otherwise, AKT, as an inhibitor of autophagy factor, could inhibit autophagy with its activation [25]. To further understand whether **2d** still affected autophagy after structure modification, the inhibition of activation AKT and phosphorylation of JNK and PI3KIII proteins were also carried out using BBR as a reference [4]. As shown in Figure 4, compound **2d** could activate AKT and inhibit the phosphorylation of JNK and PI3KIII proteins similar to BBR as reported before.

**Figure 5:**
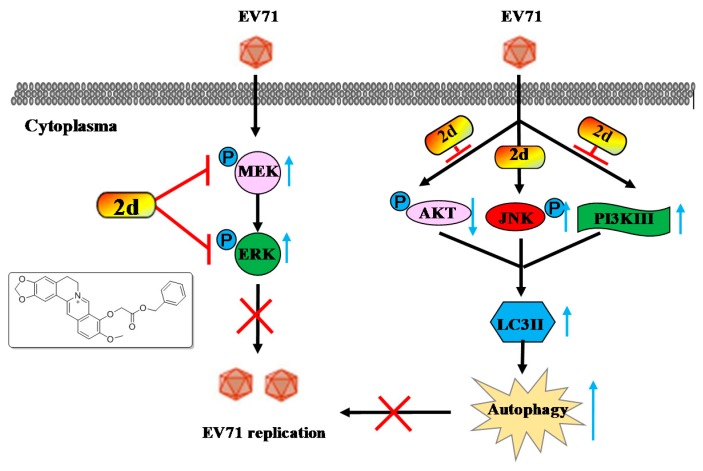
The cartoon chart of mechanism of compound **2d** against EV71.

Accordingly, we found that the lapidated LC3II, a marker for autophagy, decreased in the presence of compound **2d**, while compound **2d** could not affect the expression of Beclin-1, similar to that of BBR. Therefore, compound **2d** exerted a potent anti-EV71 effect through the same mechanism with parent BBR after the structure modification. Collectively, as depicted in the cartoon chart (Figure 5), compound **2d** might exert a potent anti-EV71 activity through inhibiting the activation of MEK/ERK pathway and suppressing EV71-induced autophagy by activating AKT and inhibiting the phosphorylation of JNK and PI3KIII. As a result, compound **2d** owned a potent anti-EV71 effect through the same action mode with parent BBR after the structure modification.

## 3. Experimental Section

### 3.1. Apparatus, Materials, and Analysis Reagents

Melting points (mps) were obtained with a CXM-300 melting point apparatus (Shanghai Changfang Optical Instrument Co., LTD., Shanghai, China) and are uncorrected. The ^1^H-NMR spectra was recorded on an Inova 500 or 600 MHz spectrometer (Varian, San Francisco, CA, USA) and ^13^C-NMR on an Avance III 400, 500 or 600 spectrometer (Bruker, Zürich, Switzerland) with Me_4_Si as the internal standard, all the samples were dissolved in DMSO-*d*_6_ before testing. High resolution mass spectra (HRMS-ESI) data was recorded on an Autospec UItima-TOF mass spectrometer (Micromass UK Ltd., Manchester, UK). Flash chromatography was performed on CombiflashRf 200 (Teledyne, Lincoln, NE, USA), particle size 0.038 mm.

Vero cells were purchased from the American Type Culture Collection (ATCC, Rockefeller, MD, USA), and were cultured in Minimum Essential Medium (MEM) supplemented with 10% fetal bovine serum (FBS) (GIBCO, Grand Island, NY, USA) and antibiotics (100 U/mL penicillin and 100 mg/mL streptomycin) at 37 °C in a 5% CO_2_ incubator. EV71 strain SHZH98 isolated from the throat swab sample of an HFMD case occurring in 1998 in China was kindly provided by Dr. Qi Jin (Institute of Pathogen Biology, Chinese Academy of Medical Science and Peking Union Medical School, Beijing, China), and JS-52 strain was a kind gift from Dr. Xiangzhong Ye (Beijing Wantai Biological Pharmacy Enterprise Co., Ltd., Beijing, China). The EV71 BrCr and H strains were purchased from the ATCC. All of EV71 strains were passaged in Vero cells.

### 3.2. Chemistry

#### 3.2.1. General Procedure for the Synthesis of Compounds **2a**–**i**

BBR (1.86 g, 5 mmol) was heated at 195–210 °C for 10–15 min under vacuum (30–40 mmHg) to afford a black oil, which was acidified with ethanol/concentrated HCl (95:5). The solvent was removed by evaporation, the residue was collected and then purified by flash chromatography over silica gel using CH_2_Cl_2_/CH_3_OH as the gradient eluent, affording the title compound **1** (1.43 g, 80%) as a yellow solid. To a stirred solution of **1** (100 mg, 0.31 mmol) in anhydrous CH_3_CN or DMF, K_2_CO_3_ (122 mg, 0.88 mmol) was added and heated to 70 °C. Then the R_1_Br or R_2_Cl (2–4.8 eq) was added and stirred for 5–6 h. The mixture was cooled to precipitate completely, filtrated and then purified by flash chromatography over silica gel using CH_2_Cl_2_/CH_3_OH as the gradient eluent to afford compounds **2a**–**i**.

*2,3-Methylenedioxy-9-ethyloxyformylmethylenoxy-10-methoxy protoberberine chloride* (**2a**). Compound **1** (1.5 g, 4.2 mmol) was treated with ethyl bromoacetate (2.22 mL, 20 mmol) according to the general procedure to give the desired product **2a**. Yield: 58%; yellow solid; m.p. 220–222 °C; ^1^H-NMR (400 MHz) δ 9.95 (s, 1H), 8.95 (s, 1H), 8.20 (d, *J* = 8.0 Hz, 1H), 8.00 (d, *J* = 8.0 Hz, 1H), 7.81 (s, 1H), 7.10 (s, 1H), 6.18 (s, 2H), 5.08 (d, *J* = 4.0 Hz, 2H), 4.94 (t, *J* = 8.0 Hz, 2H), 4.18 (q, *J* = 16.0, 2H), 4.04 (s, 3H), 3.22 (t, *J* = 8.0 Hz, 2H), 1.21 (t, *J* = 8.0 Hz, 3H). ^13^C-NMR (101 MHz) δ 169.2, 149.8, 149.2, 147.6, 145.6, 137.5, 132.8, 130.6, 126.6, 123.5, 120.3, 120.0, 108.3, 105.4, 102.0, 69.2, 69.1, 60.7, 57.1, 55.3, 51.8, 26.3, 14.0. HRMS: calcd. for C_23_H_22_ClNO_6_ [M]^+^: 408.1442, found: 408.1444.

*2,3-Methylenedioxy-9-isobutyloxyformylmethylenoxy-10-methoxy protoberberine chloride* (**2b**). Compound **1** (1.5 g, 4.2 mmol) was treated with isobutyl bromoacetate (2.93 mL, 20 mmol) according to the general procedure to give the desired product **2b**. Yield: 55%; yellow solid; m.p. 232–234 °C; ^1^H-NMR (400 MHz) δ 9.93 (s, 1H), 8.95 (s, 1H), 8.20 (d, *J* = 4.0 Hz, 1H), 7.97 (d, *J* = 4.0 Hz, 1H), 7.80 (s, 1H), 7.10 (s, 1H), 6.18 (s, 2H), 4.97 (s, 2H), 4.94 (t, *J* = 4.0 Hz, 2H), 4.08 (d, *J* = 12.0 Hz, 1H), 4.04 (s, 2H), 3.31 (s, 3H), 3.21 (t, *J* = 4.0 Hz, 2H), 1.40 (t, *J* = 7.2 Hz, 6H). ^13^C-NMR (101 MHz) δ 167.7, 149.8, 148.7, 147.6, 145.5, 141.5, 137.5, 132.9, 130.6, 126.8, 123.0, 120.9, 120.3, 120.0, 108.3, 105.4, 102.0, 81.6, 69.5, 61.8, 57.1, 55.3, 27.6 (2), 26.3. HRMS: calcd. for C_25_H_26_ClNO_6_ [M]^+^: 436.1755, found: 436.1760.

*2,3-Methylenedioxy-9-pivalylmethylenoxy-10-methoxy protoberberine chloride* (**2c**). Compound **1** (357 mg, 1 mmol) was treated with 1-bromo-3,3-dimethyl-2-butanone (358.1 mg, 2 mmol) according to the general procedure to give the desired product **2c**. Yield: 41%; yellow solid; m.p. 242–244 °C; ^1^H-NMR (500 MHz) δ 9.94 (s, 1H), 8.94 (s, 1H), 8.16 (d, *J* = 10.0 Hz, 1H), 7.95 (d, *J* = 10.0 Hz, 1H), 7.81 (s, 1H), 7.10 (s, 1H), 6.18 (s, 2H), 5.51 (s, 2H), 4.95 (t, *J* = 5.0 Hz, 2H), 4.01 (s, 3H), 3.22 (t, *J* = 5.0 Hz, 2H), 1.15 (s, 9H). ^13^C-NMR (126 MHz) δ 209.9, 149.7, 148.7, 147.6, 145.7, 141.9, 137.3, 132.8, 130.6, 126.5, 122.6, 121.0, 120.3, 119.9, 108.3, 105.3, 102.0, 73.1, 56.9, 55.3, 42.1, 26.3, 25.7 (3). HRMS: calcd. for C_25_H_26_ClNO_5_ [M]^+^: 420.1806, found: 420.1807.

*2,3-Methylenedioxy-9-benzyloxyformylmethylenoxy-10-methoxyprotoberberine chloride* (**2d**). Compound **1** (1.5 g, 4.2 mmol) was treated with benzyl bromoacetate (3.17 mL, 20 mmol) according to the general procedure to give the desired product **2d**. Yield: 58%; yellow solid; m.p. 206–208 °C; ^1^H-NMR (400 MHz) δ 9.90 (s, 1H), 8.92 (s, 1H), 8.19 (d, *J* = 8.0 Hz, 1H), 7.99 (d, *J* = 8.0 Hz, 1H), 7.80 (s, 1H), 7.34–7.29 (m, 5H), 7.09 (s, 1H), 6.18 (s, 2H), 5.19 (s, 2H), 5.16 (s, 2H), 4.84 (t, *J* = 4.0 Hz, 2H), 3.99 (s, 3H), 3.17 (t, *J* = 4.0 Hz, 2H). ^13^C-NMR (101 MHz) δ 168.7, 149.8, 149.0, 147.6, 145.6, 141.3, 137.4, 135.3, 132.8, 130.5, 128.3 (2),128.1 (3), 126.6, 123.4, 121.1, 120.3, 120.0, 108.3, 105.3, 102.0, 69.2, 66.1, 57.0, 55.2, 26.2. HRMS: calcd. for C_28_H_24_ClNO_6_ [M]^+^: 470.1598, found: 470.1598.

*2,3-Methylenedioxy-9-carboxylmethylenoxy-10-methoxy protoberberine chloride* (**2d-a**). Compound **2d** (0.5 g, 1.1 mmol) was treated with concentrated hydrochloric acid (5.0 mL) at 50 °C to give the desired product **2d-a**. Yield: 78%; yellow solid; m.p. 232–234 °C; ^1^H-NMR (600 MHz) δ 13.12 (br, 1H), 9.95 (s, 1H), 8.94 (s, 1H), 8.20 (d, *J* = 6.0 Hz, 1H), 7.99 (d, *J* = 6.0 Hz, 1H), 7.80 (s, 1H), 7.10 (s, 1H), 6.18 (s, 2H), 4.98 (s, 2H), 4.93 (t, *J* = 6.0 Hz, 2H), 4.04 (s, 3H), 3.21 (t, *J* = 6.0 Hz, 2H). ^13^C-NMR (151 MHz) δ 170.1, 149.8, 149.2, 147.6, 145.8, 141.7, 137.4, 132.9, 130.6, 126.7, 123.2, 121.2, 120.3, 120.0, 108.3, 105.4, 102.0, 69.1, 57.1, 55.3, 26.3. HRMS: calcd. for C_2__1_H_18_ClNO_6_ [M]^+^: 380.1129, found: 380.1132.

*2,3-Methylenedioxy-9-p-tolylformylmethylenoxy-10-methoxy protoberberine chloride* (**2e**). Compound **1** (357 mg, 1 mmol) was treated with 2-bromo-4′-methyl acetophenone (426.1 mg, 2 mmol) according to the general procedure to give the desired product **2e**. Yield: 42%; yellow solid; m.p. 219–221 °C; ^1^H-NMR (500 MHz) δ 10.01 (s, 1H), 8.96 (s, 1H), 8.18 (d, *J* = 5.0 Hz, 1H), 7.98 (d, *J* = 10.0 Hz, 1H), 7.82 (s, 1H), 7.60–7.58 (m, 1H), 7.52–7.47 (m, 2H), 7.29–7.27 (m, 1H), 7.11 (s, 1H), 6.19 (s, 2H), 5.89 (s, 2H), 4.96 (t, *J* = 5.0 Hz, 2H), 3.96 (s, 3H), 3.83 (s, 3H), 3.23 (t, *J* = 5.0 Hz, 2H). ^13^C-NMR (126 MHz) δ 194.2, 159.4, 149.7, 148.9, 147.6, 145.7, 141.9, 137.4, 135.2, 132.9, 130.6, 130.1, 126.6, 122.9, 121.0, 120.3, 120.1, 120.0, 119.8, 112.2, 108.3, 105.4, 102.0, 74.8, 57.0, 55.3, 55.3, 26.3. HRMS: calcd. for C_28_H_24_ClNO_5_ [M]^+^: 454.1649, found: 454.1653.

*2,3-Methylenedioxy-9-m-methoxyphenylformylmethylenoxy-10-methoxy protoberberine chloride* (**2f**). Compound **1** (357 mg, 1 mmol) was treated with 2-bromo-3′-methoxyacetophenone (458.1 mg, 2 mmol) according to the general procedure to give the desired product **2f**. Yield: 41%; yellow solid; m.p. 214–216 °C; ^1^H-NMR (500 MHz) δ 10.02 (s, 1H), 8.95 (s, 1H), 8.17 (d, *J* = 10.0 Hz, 1H), 7.97 (d, *J* = 10.0 Hz, 1H), 7.89 (d, *J* = 10.0 Hz, 2H), 7.82 (s, 1H), 7.38 (d, *J* = 10.0 Hz, 2H), 7.11 (s, 1H), 6.18 (s, 2H), 5.87 (s, 2H), 4.95 (t, *J* = 5.0 Hz, 2H), 3.94 (s, 3H), 3.22 (t, *J* = 5.0 Hz, 2H), 2.40 (s, 3H). ^13^C-NMR (126 MHz) δ 193.8, 149.7, 148.9, 147.6, 145.8, 144.4, 142.0, 137.4, 132.9, 131.4, 130.6, 129.4 (2), 127.7 (2), 126.6, 122.8, 121.0, 120.3, 120.0, 108.3, 105.3, 102.0, 74.6, 57.0, 55.3, 26.3, 21.2. HRMS: calcd. for C_28_H_24_ClNO_6_ [M]^+^: 470.1598, found: 470.1598.

*2,3-Methylenedioxy-9-p-trifloromethylphenylformylmethylenoxy-10-methoxy protoberberine chloride* (**2g**). Compound **1** (357 mg, 1 mmol) was treated with 4-(trifluoromethyl)phenacyl bromide (534.1 mg, 2 mmol) according to the general procedure to give the desired product **2g**. Yield: 40%; yellow solid; m.p. 238–240 °C; ^1^H-NMR (500 MHz) δ 10.02 (s, 1H), 8.97 (s, 1H), 8.20 (t, *J* = 10.0 Hz, 3H), 7.98 (t, *J* = 10.0 Hz, 3H), 7.82 (s, 1H), 7.11 (s, 1H), 6.19 (s, 2H), 5.93 (s, 2H), 4.96 (t, *J* = 5.0 Hz, 2H), 3.94 (s, 3H), 3.23 (t, *J* = 5.0 Hz, 2H). ^13^C-NMR (126 MHz) δ 194.0, 149.8, 148.9, 147.6, 145.7, 141.7, 137.5, 137.2, 133.1, 132.9, 132.9, 130.6, 128.6 (3), 126.6, 125.8, 123.1, 120.9, 120.3, 120.0, 108.4, 105.4, 102.0, 75.0, 57.0, 55.3, 26.3. HRMS: calcd. for C_28_H_21_ClF_3_NO_5_ [M]^+^: 508.1366, found: 508.1366.

*2,3-Methylenedioxy-9-benzyloxyformyloxy-10-methoxy protoberberine chloride* (**2h**). Compound **1** (357 mg, 1 mmol) was treated with benzyl chloroformate (682.4 mg, 4 mmol) according to the general procedure to give the desired product **2h**. Yield: 38%; yellow solid; m.p. 195–197 °C; ^1^H-NMR (400 MHz) δ 10.03 (s, 1H), 9.06 (s, 1H), 8.31 (d, *J* = 10.0 Hz, 1H), 8.24 (d, *J* = 15.0 Hz, 1H), 7.82 (s, 1H), 7.52–7.44 (m, 5H), 7.11 (s, 1H), 6.19 (s, 2H), 5.41 (s, 2H), 4.94 (t, *J* = 10.0 Hz, 2H), 4.02 (s, 3H), 3.22 (t, *J* = 10.0 Hz, 2H). ^13^C-NMR (101 MHz) δ 151.5, 150.2, 150.0, 147.6, 144.2, 138.2, 134.6, 133.4, 132.7, 130.8, 128.6, 128.5 (2), 128.1 (2), 127.0, 125.9, 120.5(2), 120.2, 108.3, 105.4, 102.0, 70.7, 57.2, 55.3, 26.1. HRMS: calcd. for C_27_H_22_ClNO_6_ [M]^+^: 456.1442, found: 454.1441.

*2,3-Methylenedioxy-9-ethyloxyformyloxy-10-methoxy protoberberine chloride* (**2i**). Compound **1** (1.5 g, 4.2 mmol) was treated with ethyl chloroformate (1.91 mL, 20 mmol) according to the general procedure to give the desired product **2i**. Yield: 37%; yellow solid; m.p. 218–220 °C; ^1^H-NMR (400 MHz) δ 9.99 (s, 1H), 9.06 (s, 1H), 8.32 (d, *J* = 4.0 Hz, 1H), 8.24 (d, *J* = 4.0 Hz, 1H), 7.82 (s, 1H), 7.10 (s, 1H), 6.18 (s, 2H), 4.95 (t, *J* = 4.0 Hz, 2H), 4.38 (q, *J* = 8.0 Hz, 2H), 4.08 (s, 3H), 3.22 (t, *J* = 4.0 Hz, 2H), 1.38 (t, *J* = 8.0 Hz, 3H). ^13^C-NMR (101 MHz) δ 152.0, 150.9, 150.5, 148.2, 144.8, 138.7, 133.9, 133.3, 131.4, 127.5, 126.5, 121.1(2), 120.8, 108.9, 106.0, 102.6, 66.3, 57.8, 55.8, 26.6, 14.4. HRMS: calcd. for C_22_H_20_ClNO_6_ [M]^+^: 394.1285, found: 394.1285.

#### 3.2.2. General Procedure for the Synthesis of Compounds **4a**–**g**

PMT (1.94 g, 5 mmol) was heated at 195–210 °C for 10–15 min under vacuum (30–40 mmHg) to afford the black oil, which was acidified with ethanol/concentrated HCl (95:5). The solvent was removed by evaporation, the residue was collected and then purified by flash chromatography over silica gel using CH_2_Cl_2_/CH_3_OH as the gradient eluent, affording the title compound **3** (1.6 g, 86%) as a yellow solid. To a stirred solution of compound **3** (100 mg, 0.30 mmol) in anhydrous CH_3_CN or DMF, K_2_CO_3_ (122 mg, 0.88 mmol) was added at room temperature. Then R_1_Br or benzyl chloroformate (2–4 eq) was added and stirred for 0.5–1 h. The mixture was cooled to precipitate completely, filtrated and washed by CH_2_Cl_2_ to afford compounds **4a**–**g**. The final products were purified by flash chromatography over silica gel using CH_2_Cl_2_/CH_3_OH as the gradient eluent.

*2,3,10-Trimethoxy-9-ethyloxyformylmethylenoxy protoberberine chloride* (**4a**). Compound **3** (373 mg, 1 mmol) was treated with ethyl bromoacetate (668 mg, 4 mmol) according to the general procedure to give the desired product **4a**. Yield: 24%; yellow solid; m.p. 204–206 °C; ^1^H-NMR (500 MHz) δ 9.95 (s, 1H), 9.05 (s, 1H), 8.21 (d, *J* = 10.0 Hz, 1H), 8.03 (d, *J* = 10.0 Hz, 1H), 7.73 (s, 1H), 7.11 (s, 1H), 5.07 (s, 2H), 4.96 (t, *J* = 5.0 Hz, 2H), 4.18 (dd, *J* = 5.0 Hz, *J* = 10.0 Hz, 2H), 4.04 (s, 3H), 3.94 (s, 3H), 3.88 (s, 3H), 3.24 (t, *J* = 10.0 Hz, 2H), 1.21 (t, *J* = 10.0 Hz, 3H). ^13^C-NMR (126 MHz) δ 168.8, 151.4, 148.9, 148.6, 145.7, 141.4, 137.7, 132.9, 128.5, 126.7, 123.2, 121.0, 119.7, 118.8, 111.2, 108.6, 69.2, 60.7, 57.0, 56.0, 55.8, 55.5, 25.9, 14.0. HRMS: calcd. for C_24_H_26_ClNO_6_ [M]^+^: 424.1755, found: 424.1757.

*2,3,10-Trimethoxy-9-pivalylmethylenoxy protoberberine chloride* (**4b**). Compound **3** (500 mg, 1.34 mmol) was treated with 1-bromo-3,3-dimethyl-2-butanone (479.9 mg, 2.68 mmol) according to the general procedure to give the desired product **4b**. Yield: 25%; yellow solid; m.p. 227–229 °C; ^1^H-NMR (500 MHz) δ 9.94 (s, 1H), 9.03 (s, 1H), 8.17 (d, *J* = 10.0 Hz, 1H), 7.98 (d, *J* = 10.0 Hz, 1H), 7.73 (s, 1H), 7.11 (s, 1H), 5.51 (s, 2H), 4.97 (t, *J* = 5.0 Hz, 2H), 4.02 (s, 3H), 3.94 (s, 3H), 3.88 (s, 3H), 3.24 (t, *J* = 5.0 Hz, 2H), 1.15 (s, 9H). ^13^C-NMR (126 MHz) δ 209.9, 151.3, 148.6 (2), 145.7, 141.9, 137.5, 132.9, 128.5, 126.5, 122.5, 120.9, 119.6, 118.8, 111.1, 108.6, 73.1, 56.9, 56.0, 55.7, 55.4, 42.1, 25.9, 25.7 (3). HRMS: calcd. for C_26_H_30_ClNO_5_ [M]^+^: 436.2119, found: 436.2119.

*2,3,10-Trimethoxy-9-benzyloxyformylmethylenoxy protoberberine chloride* (**4c**). Compound 3 (373 mg, 1 mmol) was treated with benzyl bromoacetate (916.2 mg, 4 mmol) according to the general procedure to give the desired product **4c**. Yield: 29%; yellow solid; m.p. 196–198 °C; ^1^H-NMR (500 MHz) δ 9.90 (s, 1H), 9.01 (s, 1H), 8.20 (d, *J* = 9.2 Hz, 1H), 8.02 (d, *J* = 9.1 Hz, 1H), 7.71 (s, 1H), 7.32 (m, 5H), 7.10 (s, 1H), 5.18 (d, *J* = 15.1 Hz, 4H), 4.85 (t, *J* = 6.4 Hz, 2H), 3.99 (s, 3H), 3.95 (s, 3H), 3.88 (s, 3H), 3.19 (t, *J* = 6.4 Hz, 2H). ^13^C-NMR (126 MHz) δ 168.7, 151.4, 148.8, 148.6, 145.6, 141.3, 137.6, 135.3, 132.9, 128.5, 128.3 (2), 128.1 (2), 126.6, 123.2, 121.0, 119.6, 118.7, 111.1, 108.5, 69.2, 66.1, 57.0, 56.0, 55.7, 55.3, 25.9. HRMS: calcd. for C_29_H_28_ClNO_6_ [M]^+^: 486.1911, found: 486.1916.

*2,3,10-Trimethoxy-9-p-tolylformylmethylenoxy protoberberine chloride* (**4d**). Compound **3** (500 mg, 1.34 mmol) was treated with 2-bromo-4′-methyl acetophenone (571.0 mg, 2.68 mmol) according to the general procedure to give the desired product **4d**. Yield: 21%; yellow solid; m.p. 228–230 °C; ^1^H-NMR (500 MHz) δ 10.02 (s, 1H), 9.05 (s, 1H), 8.18 (d, *J* = 10.0 Hz, 1H), 8.01 (d, *J* = 10.0 Hz, 1H), 7.90 (d, *J* = 10.0 Hz, 2H), 7.74 (s, 1H), 7.39 (d, *J* = 10.0 Hz, 2H), 7.11 (s, 1H), 5.87 (s, 2H), 4.97 (t, *J* = 5.0 Hz, 2H), 3.95 (d, *J* = 5.0 Hz, 6H), 3.88 (s, 3H), 3.25 (t, *J* = 5.0 Hz, 2H), 2.40 (s, 3H). ^13^C-NMR (126 MHz) δ 194.5, 151.9, 149.3, 149.2, 146.4, 144.9, 142.5, 138.2, 133.6, 132.0, 130.0 (2), 129.1, 128.3 (2), 127.2, 123.3, 121.5, 120.2, 119.4, 111.7, 109.2, 75.2, 57.6, 56.6, 56.3, 56.0, 26.5, 21.7. HRMS: calcd. for C_29_H_28_ClNO_5_ [M]^+^: 470.1962, found: 470.1961.

*2,3,10-Trimethoxy-9-m-methoxyphenylformylmethylenoxy protoberberine chloride* (**4e**). Compound **3** (500 mg, 1.34 mmol) was treated with 2-bromo-3′-methoxyacetophenone (613.9 mg, 2.68 mmol) according to the general procedure to give the desired product **4e**. Yield: 20%; red brown solid; m.p. 200–202 °C; ^1^H-NMR (400 MHz) δ 10.02 (s, 1H), 9.07 (s, 1H), 8.18 (d, *J* = 10.0 Hz, 1H), 8.02 (d, *J* = 10.0 Hz, 1H), 7.74 (s, 1H), 7.60 (d, *J* = 10.0 Hz, 1H), 7.52–7.47 (m, 2H), 7.30–7.27 (m, 1H), 7.12 (s, 1H), 5.89 (s, 2H), 4.98 (t, *J* = 5.0 Hz, 2H), 3.95 (s, 6H), 3.88 (s, 3H), 3.83 (s, 3H), 3.24 (t, *J* = 5.0 Hz, 2H). ^13^C-NMR (101 MHz) δ 194.2, 159.4, 151.4, 148.8, 148.6, 145.7, 141.9, 137.6, 135.3, 133.0, 130.1, 128.5, 126.7, 122.8, 121.0, 120.1, 119.8, 119.7, 118.8, 112.2, 111.2, 108.7, 74.8, 57.0, 56.1, 55.8, 55.5, 55.3, 26.0. HRMS: calcd. for C_29_H_28_ClNO_6_ [M]^+^: 486.1911, found: 486.1916.

*2,3,10-Trimethoxy-9-p-trifloromethylphenylformylmethylenoxy protoberberine chloride* (**4f**). Compound **3** (373 mg, 1 mmol) was treated with 4-(trifluoromethyl) phenacyl bromide (534 mg, 2 mmol) according to the general procedure to give the desired product **4f**. Yield: 29%; red brown solid; m.p. 240–242 °C; ^1^H-NMR (400 MHz) δ 10.01 (s, 1H), 9.05 (s, 1H), 8.21–8.18 (m, 3H), 8.03–7.96 (m, 3H), 7.74 (s, 1H), 7.12 (s, 1H), 5.93 (s, 2H), 4.97 (t, *J* = 5.0 Hz, 2H), 3.95 (d, *J* = 5.0 Hz, 6H), 3.88 (s, 3H), 3.25 (t, *J* = 5.0 Hz, 2H). ^13^C-NMR (101 MHz) δ 194.0, 151.4, 148.8, 148.6, 145.7, 141.7, 137.7, 137.3, 133.0, 128.6 (2), 128.5, 126.7, 125.8 (2), 125.3, 124.9, 122.94, 120.9, 119.77, 118.7, 111.2, 108.7, 75.0, 57.0, 56.1, 55.8, 55.5, 25.9. HRMS: calcd. for C_29_H_25_ClF_3_NO_5_ [M]^+^: 524.1679, found: 524.1686.

*2,3,10-Trimethoxy-9-benzyloxyformyloxy protoberberine chloride* (**4g**). Compound **3** (373 mg, 1 mmol) was treated with benzyl chloroformate (682.4 mg, 4 mmol) according to the general procedure to give the desired product **4g**. Yield: 21%; yellow solid; m.p. 98–100 °C; ^1^H-NMR (400 MHz) δ 10.02 (s, 1H), 9.16 (s, 1H), 8.29 (dd, *J* = 10.0 Hz, *J* = 25.0 Hz, 2H), 7.74 (s, 1H), 7.53–7.43 (m, 5H), 7.11 (s, 1H), 5.41 (s, 2H), 4.96 (t, *J* = 5.0 Hz, 2H), 4.03 (s, 3H), 3.95 (s, 3H), 3.88 (s, 3H), 3.24 (t, *J* = 5.0 Hz, 2H). ^13^C-NMR (101 MHz) δ 151.6, 150.0, 148.6, 144.2, 138.4, 134.6, 133.3, 132.8, 128.7, 128.6, 128.5 (2), 128.1 (2), 126.9, 125.9, 120.4, 120.2, 118.7, 111.2, 108.7, 70.7, 57.2, 56.1, 55.8, 55.4, 45.3, 25.7. HRMS: calcd. for C_28_H_26_ClNO_6_ [M]^+^: 472.1755, found: 472.1758.

#### 3.2.3. General Procedure for the Synthesis of Compounds **5a**–**g**

To a stirred solution of JTH (100 mg, 0.29 mmol) in anhydrous CH_3_CN or DMF, K_2_CO_3_ (122 mg, 0.88 mmol) was added and heated to 70 °C. Then R_1_Br or benzyl chloroformate (2–4 eq) was added and stirred for 5–6 h. The mixture was cooled to precipitate completely, filtrated and washed by CH_2_Cl_2_ to afford compounds **5a**–**g**.

*2,9,10-Trimethoxy-3-ethyloxyformylmethylenoxy protoberberine chloride* (**5a**). JTH (373 mg, 1 mmol) was treated with ethyl bromoacetate (668 mg, 4 mmol) according to the general procedure to give the desired product **5a**. Yield: 35%; yellow solid; m.p. 234–236 °C; ^1^H-NMR (500 MHz) δ 9.91 (s, 1H), 9.07 (s, 1H), 8.23 (d, *J* = 10.0 Hz, 1H), 8.06–8.04 (m, 1H), 7.76 (s, 1H), 7.04 (s, 1H), 4.94 (t, *J* = 5.0 Hz, 2H), 4.92 (s, 2H), 4.20 (dd, *J* = 5.0 Hz, *J* = 15.0 Hz, 2H), 4.10 (s, 3H), 4.08 (s, 3H), 3.97 (s, 3H), 3.19 (t, *J* = 5.0 Hz, 2H), 1.24 (t, *J* = 5.0 Hz, 3H). ^13^C-NMR (126 MHz) δ 168.2, 150.2, 149.5, 148.6, 145.4, 143.5, 137.4, 132.9, 128.2, 126.6, 123.3, 121.3, 112.0, 119.7, 112.4, 109.1, 64.9, 61.8, 60.7, 56.9, 56.1, 55.2, 25.7, 14.0. HRMS: calcd. for C_24_H_26_ClNO_6_ [M]^+^: 424.1755, found: 424.1751.

*2,9,10-Trimethoxy-3-pivalylmethylenoxy protoberberine chloride* (**5b**). JTH (373 mg, 1 mmol) was treated with 1-bromo-3,3-dimethyl-2-butanone (358.1 mg, 2 mmol) according to the general procedure to give the desired product **5b**. Yield: 28%; yellow solid; m.p. 223–225 °C; ^1^H-NMR (500 MHz) δ 9.90 (s, 1H), 9.05 (s, 1H), 8.23 (d, *J* = 5.0 Hz, 1H), 8.05 (d, *J* = 5.0 Hz, 1H), 7.74 (s, 1H), 6.88 (s, 1H), 5.27 (s, 2H), 4.94 (t, *J* = 5.0 Hz, 2H), 4.10 (s, 3H), 4.08 (s, 3H), 3.96 (s, 3H), 3.18 (t, *J* = 5.0 Hz, 2H), 1.20 (s, 9H). ^13^C NMR (126 MHz) δ 209.2, 150.2, 145.0, 148.6, 145.3, 143.5, 137.5, 132.9, 128.1, 126.6, 123.3, 121.2, 119.8, 119.1, 112.0, 109.1, 68.8, 61.8, 56.9, 56.1, 55.2, 42.4, 25.8, 25.6 (3). ^13^C-NMR (126 MHz) δ 209.2, 150.2, 145.0, 148.6, 145.3, 143.5, 137.5, 132.9, 128.1, 126.6, 123.3, 121.2, 119.8, 119.1, 112.0, 109.1, 68.8, 61.8, 56.9, 56.1, 55.2, 42.4, 25.8, 25.6 (3). HRMS: calcd. for C_26_H_30_ ClNO_5_ [M]^+^: 436.2119, found: 436.2118.

*2,9,10-Trimethoxy-3-benzyloxyformylmethylenoxy protoberberine chloride* (**5c**). JTH (373 mg, 1 mmol) was treated with benzyl bromoacetate (916.2 mg, 4 mmol) according to the general procedure to give the desired product **5c**. Yield: 38%; yellow solid; m.p. 217–219 °C; ^1^H-NMR (500 MHz) δ 9.90 (s, 1H), 9.06 (s, 1H), 8.23 (d, *J* = 10.0 Hz, 1H), 8.04 (d, *J* = 10.0 Hz, 1H), 7.75 (s, 1H), 7.40–7.35 (m, 4H), 7.03 (s, 1H), 5.23 (s, 2H), 5.01 (s, 2H), 4.93 (t, *J* = 5.0 Hz, 2H), 4.10 (s, 3H), 4.08 (s, 3H), 3.96 (s, 3H), 3.16 (t, *J* = 5.0 Hz, 2H). ^13^C-NMR (126 MHz) δ 168.2, 150.2, 149.5, 148.6, 145.4, 143.5, 137.3, 135.5, 132.9, 128.34 (2), 128.1, 128.1, 127.94 (2), 126.6, 123.3, 121.3, 120.0, 119.73, 112.4, 109.2, 66.0, 64.9, 61.8, 56.9, 56.1, 55.2, 25.8. HRMS: calcd. for C_29_H_28_ClNO_6_ [M]^+^: 486.1911, found: 486.1910.

*2,9,10-Trimethoxy-3-p-methylphenylformylmethylenoxy protoberberine chloride* (**5d**). JTH (373 mg, 1 mmol) was treated with 2-bromo-4′-methylacetophenone (426.1 mg, 2 mmol) according to the general procedure to give the desired product **5d**. Yield: 38%; yellow solid; m.p. 218–220 °C; ^1^H-NMR (500 MHz) δ 9.89 (s, 1H), 9.06 (s, 1H), 8.23 (d, *J* = 10.0 Hz, 1H), 8.05 (d, *J* = 10.0 Hz, 1H), 7.76 (s, 1H), 7.65–7.63 (m, 1H), 7.54–7.51 (m, 2H), 7.32–7.29 (m, 1H), 7.06 (s, 1H), 5.75 (s, 2H), 4.93 (t, *J* = 5.0 Hz, 2H), 4.10 (s, 3H), 4.08 (s, 3H), 3.99 (s, 3H), 3.85 (s, 3H), 3.16 (t, *J* = 5.0 Hz, 2H). ^13^C-NMR (126 MHz) δ 193.5, 159.3, 150.2, 150.0, 148.6, 145.3, 143.5, 137.5, 135.4, 132.9, 130.0, 128.2, 126.6, 123.3, 121.3, 120.2, 119.8, 119.6, 119.2, 112.5, 112.3, 109.1, 70.5, 61.8, 56.9, 56.1, 55.4, 55.2, 25.7. HRMS: calcd. for C_29_H_28_ClNO_5_ [M]^+^: 470.1962, found: 470.1965.

*2,9,10-Trimethoxy-3-m-methoxyphenylformylmethylenoxy protoberberine chloride* (**5e**). JTH (373 mg, 1 mmol) was treated with 2-bromo-3′-methoxyacetophenone (458.1 mg, 2 mmol) according to the general procedure to give the desired product **5e**. Yield: 36%;yellow solid; m.p. 218–220 °C; ^1^H-NMR (500 MHz) δ 9.89 (s, 1H), 9.06 (s, 1H), 8.23 (d, *J* = 10.0 Hz, 1H), 8.05 (d, *J* = 10.0 Hz, 1H), 7.96–7.94 (m, 2H), 7.76 (s, 1H), 7.42–7.40 (m, 2H), 7.04 (s, 1H), 5.71 (s, 2H), 4.93 (t, *J* = 5.0 Hz, 2H), 4.10 (s, 3H), 4.08 (s, 3H), 3.99 (s, 3H), 3.15 (t, *J* = 5.0 Hz, 2H), 2.42 (s, 3H). ^13^C-NMR (126 MHz) δ 193.2, 150.2, 150.1, 148.6, 145.3, 144.3, 143.5, 137.5, 132.9, 131.6, 129.3 (2), 128.2, 127.9 (2), 126.6, 123.3, 121.3, 119.8, 119.2, 112.3, 109.1, 70.3, 61.8, 56.9, 56.1, 55.2, 25.7, 21.2. HRMS: calcd. for C_29_H_28_ClNO_6_ [M]^+^: 486.1911, found: 486.1914.

*2,9,10-Trimethoxy-3-p-trifloromethylphenylformylmethylenoxy protober berine chloride* (**5f**). JTH (373 mg, 1 mmol) was treated with 4-(trifluoromethyl) phenacyl bromide (534.1 mg, 2 mmol) according to the general procedure to give the desired product **5f**. Yield: 35%; yellow solid; m.p. 213–215 °C; ^1^H-NMR (500 MHz) δ 9.90 (s, 1H), 9.07 (s, 1H), 8.25–8.22 (m, 3H), 8.05 (d, *J* = 10.0 Hz, 1H), 8.01–7.99 (m, 2H), 7.77 (s, 1H), 7.13 (s, 1H), 5.81 (s, 2H), 4.93 (t, *J* = 5.0 Hz, 2H), 4.10 (s, 3H), 4.08 (s, 3H), 3.99 (s, 3H), 3.16 (t, *J* = 5.0 Hz, 2H). ^13^C-NMR (126 MHz) δ 193.3, 150.2, 149.9, 148.6, 145.4, 143.5, 137.5, 137.3, 132.9, 132.8, 128.7, 128.2, 126.6, 125.7 (2), 124.7, 123.3, 122.5, 121.3, 119.9, 119.4, 112.4, 109.1, 70.7, 61.8, 56.9, 56.1, 55.2, 25.7. HRMS: calcd. for C_29_H_25_ClF_3_NO_5_ [M]^+^: 524.1679, found: 524.1679.

*2,9,10-Trimethoxy-3-benzyloxyformyloxy protoberberine chloride* (**5g**). JTH (373 mg, 1 mmol) was treated with benzyl chloroformate (682.4 mg, 4 mmol) according to the general procedure to give the desired product **5g**. Yield: 36%; yellow solid; m.p. 140–142 °C; ^1^H-NMR (500 MHz) δ 9.99 (s, 1H), 9.22 (s, 1H), 8.27 (d, *J* = 10.0 Hz, 1H), 8.10 (d, *J* = 10.0 Hz, 1H), 7.95 (s, 1H), 7.46–7.41 (m, 6H), 5.32 (s, 2H), 4.99 (t, *J* = 5.0 Hz, 2H), 4.12 (s, 3H), 4.09 (s, 3H), 3.97 (s, 3H), 3.24 (t, *J* = 5.0 Hz, 2H). ^13^C-NMR (126 MHz) δ152.1, 150.8, 150.7, 145.9, 143.7, 141.3, 136.5, 134.8, 132.5, 128.5, 128.5(2), 128.2(2), 127.9, 126.6, 125.7, 123.6, 122.3, 121.7, 121.4, 110.1, 70.0, 61.9, 56.9, 56.5, 55.2, 25.3. HRMS: calcd. for C_28_H_26_ClNO_6_ [M]^+^: 472.1755, found: 472.1753.

### 3.3. Biology Assays

#### 3.3.1. CPE Inhibition Assay for Anti-EV71

The anti-EV71 activities of all tested compounds were detected by the virus-induced CPE assay. Briefly, cells (3 × 10^4^ cells/well) were plated into 96-well culture plates and incubated for 16 h. Then, remove the medium and infected cells with EV71 of 100 × TCID_50_ (50% tissue culture infective doses) in serum-free medium for 1 h at 37 °C. After that, the unbound viruses were removed and various concentrations of tested compounds were supplemented for incubation of another 72 h. IC_50_ defined as the minimal concentration required to inhibit 50% of CPE was determined by Reed & Muench method in 48 h. TC_50_ defined as the concentration that leads to the 50% of CPE which determined by the same method in 72 h. In addition, the cells were stained the cells were colored with 0.5% crystal violet in 20% ethanol for 15 min at room temperature and the cells were imaged after rinsed with PBS.

#### 3.3.2. Cytotoxicity Assay

Cytotoxicity of all target compounds in Vero cells was analyzed by CCK (TransGen Biotech, Beijing, China) assay. In brief, cells in exponential phase (3 × 10^4^ cells/well) were seeded into 96-well culture plates and incubated overnight. Then, the medium was removed and different concentrations of all target compounds were applied in duple. After 48 h incubation, the cytotoxicity of all target compounds was determined by CCK assay. The signals were read at 450 nm on Enspire (Perkin Elmer, Waltham, MA, USA). The TC_50_ was defined as the concentration that inhibits 50% cellular growth in comparison with the controls. It is calculated by the Reed and Muench method.

#### 3.3.3. Western Blot Analysis

Cells were lysed in the M-PER mammalian protein extraction reagent (Thermo, Rockford, IL, USA) containing halt protease inhibitor single-use cocktail (Thermo). The protein concentration was determined by BCA Protein Assay Kit (Thermo). Equal amount of samples (15 μg proteins) were denatured and applied to sodium dodecyl sulfate-polyacrylamide gel electrophoresis (SDS-PAGE), and then electrophoresis products were transferred to a polyvinylidenefluoride (PVDF) film and blocked by 5% (*w*/*v*) milk or BSA at room temperature. An hour later, PVDF membranes were incubated at room temperature with specific primary antibody. After a standard washing, membranes were incubated with horse radish peroxidase (HRP)-labeled secondary antibody. The signals were detected using ECL detection kit (GE Healthcare Life Sciences, Pittsburgh, PA, USA). The primary antibodies used in the experiment included β-actin, p-p44/p42 MAPK, p44/p42 MAPK, p-MEK, MEK, p-JNK, JNK, p-AKT, AKT, PI3KIII, LC3B, Beclin-1 (Cell Signaling Technology, Danvers, MA, USA), and EV71-VP1 (Abnova, Taibei, China). HRP-labeled secondary antibodies included goat anti-rabbit and anti-mouse HRP-labeled antibodies (Cell Signaling Technology).

#### 3.3.4. qRT-PCR Quantification

Vero cells (9 × 10^5^ cells/well) were plated into 6-well culture plates and incubated for 16 h. The medium was removed and cells were infected with EV71 for 1 h (H). Various concentrations of compound **2d** and BBR were supplemented for incubation of another 24 h. Total RNA of the infected cells was isolated using the RNeasy Mini kit (QIAGEN, Hilden, Germany) and the information of the primer was listed in the Table 2.

One-step qRT-PCR was performed with SuperScript III Platinum SYBR Green One-step RT-PCR Kit (Invitrogen, Carlsbad, CA, USA) using the ABI 7500 Fast real-time PCR system (Applied Biosystems, Carlsbad, CA, USA). PCR assay was carried out in a 25 μL volume and the target fragment amplification was carried out as follows: reverse transcription at 50 °C for 3 min; initial activation of HotStar Taq DNA Polymerase at 95 °C for 10 min; 40 cycles in two steps: 95 °C for 15 s, 60 °C for 30 s.

#### 3.3.5. Statistical Analysis

Data were expressed as the mean ± standard error of the mean and analyzed with one-way ANOVA using MTLAB software (8.6, MathWorks, 2015, Natick, MA, USA). A threshold of *p* < 0.05 was defined as statistically significant.

## 4. Conclusions

Taking BBR as the lead, 23 new BBR derivatives were synthesized and examined for their anti-EV71 activities against different genotype strains with CPE assay. SAR indicated that introduction of a suitable 9-ether group might be beneficial for potency. Among them, compound **2d** exhibited most potent activities against all tested EV71 strains with IC_50_ values in the range of 7.12–14.8 μM. Its effect was further confirmed in a dose-dependent manner both in RNA and protein level, better than that of BBR. The preliminary mechanism revealed that compound **2d** could inhibit the activation of MEK/ERK signaling pathway. Furthermore, **2d** could suppress the EV71-induced autophagy by activating AKT and inhibiting the phosphorylation of JNK and PI3KIII proteins. Compound **2d** owned a potent anti-EV71 effect with new mechanism of action, has been selected for next investigation. We consider BBR derivatives to be a new class of antiviral agents against EV71 through targeting host components. The results provided the powerful information for further development of this kind of compounds into a novel family of antiviral candidates against EV71, with an advantage of broad-spectrum anti-EV71 potency.

## Figures and Tables

**Table 1:**
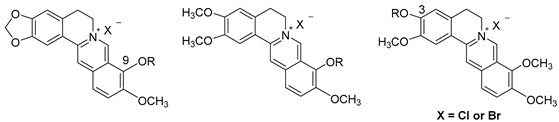
SAR of all target compounds for anti-EV71 activity and cytotoxicity in Vero cells.

No.	R	TC_50_^a^	H Strain		JS-52 Strain		SHZH98 Strain		BrCr Strain	
			IC_50_^a^	SI ^b^	IC_50_^a^	SI	IC_50_^a^	SI	IC_50_^a^	SI
BBR	-	147	21.2	6.93	14.0	10.5	28.2	5.22	9.45	15.6
**1**	-	85.5	37.0	2.31	28.5	3.00	32.6	2.62	35.4	2.41
**3**	-	254	121	2.10	102	2.50	132	1.93	135	1.87
JTH	-	255	>147	-	147	1.73	147	1.73	>147	-
**2a**	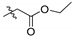	223	48.3	4.62	45.5	4.89	60.4	3.69	32.3	6.90
**2b**	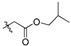	22.3	>5.34	-	5.73	3.89	5.46	4.08	2.04	10.9
**2c**	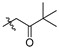	27.9	13.8	2.02	7.26	3.84	8.59	3.24	4.26	6.54
**2d**	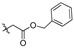	82.8	12.4	6.70	7.12	11.6	14.8	5.58	8.52	9.72
**2d-a**	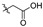	437	>145	-	145	3.00	>145	-	145	3.00
**2e**	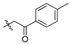	14.4	>5.31	-	2.67	5.40	2.84	5.08	2.23	6.47
**2f**	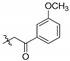	9.22	>5.33	-	1.61	5.74	1.85	4.97	2.21	4.17
**2g**	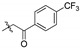	54.9	15.8	3.47	11.9	4.62	12.3	4.46	10.6	5.18
**2h**	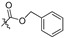	124	>47.9	-	>47.9	-	>47.9	-	47.9	2.59
**2i**	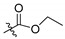	167	31.1	5.37	43.6	3.83	45.7	3.65	33.6	4.97
**4a**	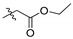	434	144	3.01	>144	-	>144	-	>144	-
**4b**	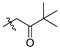	333	>144	-	109	3.05	>144	-	144	2.32
**4c**	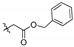	452	>143	-	99.3	4.55	>143	-	81.1	5.57
**4d**	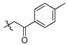	50.0	>15.9	-	44.2	1.13	>47.8	-	44.2	1.13
**4e**	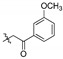	143	13.6	10.5	19.4	7.35	27.6	5.18	17.1	8.36
**4f**	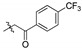	306	82.2	3.73	54.2	5.65	82.2	3.73	66.0	4.64
**4g**	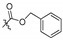	351	>143	-	129	2.72	>143	-	>143	-
**5a**	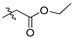	250	130	1.93	109	2.29	144	1.74	125	2.01
**5b**	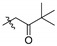	83.3	41.3	2.02	34.2	2.44	69.3	1.20	34.2	2.44
**5c**	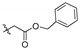	248	93.7	2.65	101	2.46	123	2.01	44.1	5.62
**5d**	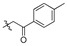	41.0	>15.9	-	9.01	4.55	12.0	3.41	>15.9	-
**5e**	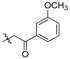	27.6	10.4	2.65	8.69	3.18	9.13	3.03	9.20	3.01
**5f**	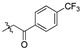	143	40.4	3.54	27.0	5.36	68.4	2.09	25.4	5.63
**5g**	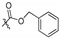	265	143	1.85	51.3	5.16	86.7	3.05	82.8	3.19

^a^ The unit of μM was used to measure TC_50_ (median toxic concentration) and IC_50_ values which are calculated based on the organic part of the target compounds. The average values of three batches were shown in this table (n = 3). ^b^ SI was calculated as the ratio of TC_50_ and IC_50_.

**Table 2:** Oligonucleotides used for real-time RT-PCR.

Oligonucleotide	Sequence (5′–3′)
5′VP1(EV71)	5′-GCAGCCCAAAAGAACTTCAC-3′
3′VP1(EV71)	5′-ATTTCAGCAGCTTGGAGTGC-3′
5′β-actin	5′-TGACGGGGTCACCCACACTGTGCCCATCTA-3′
3′β-actin	5′-CTAGAAGCATTTGCGGTGGACGATG-3′

## Supplementary Materials

The following are available online. Figure S1: ^1^H-NMR, ^13^C-NMR, HRMS-ESI spectra.

## References

[1] McMinn P.C. (2012). Recent advances in the molecular epidemiology and control of human enterovirus 71 infection. Curr. Opin. Virol..

[2] Balestri R., Bellino M., Landini L., Tasin L., Rizzoli L., Speziali L., Bauer P., Sicher M.C., Rech G., Girardelli C.R. (2014). Atypical presentation of enterovirus infection in adults: Outbreak of “Hand, Foot, Mouth and Scalp disease” in Northern Italy. J. Eur. Acad. Dermatol. Venereol..

[3] Weng K.F., Chen L.L., Huang P.N., Shih S.R. (2010). Neural pathogenesis of enterovirus 71 infection. Microbes Infect..

[4] Wang H.Q., Li K., Ma L.L., Wu S., Hu J., Yan H.Y., Jiang J.D., Li Y.H. (2017). Berberine inhibits enterovirus 71 replication by downregulating the MEK/ERK signaling pathway and autophagy. Virol. J..

[5] Chang L.Y., Lin T.Y., Hsu K.H., Huang Y.C., Lin K.L., Hsueh C., Shih S.R., Ning H.C., Hwang M.S., Wang H.S. (1999). Clinical features and risk factors of pulmonary oedema after enterovirus-71-related hand, foot, and mouth disease. Lancet.

[6] Zhang H., Li F.Q., Pan Z.Y., Wu Z.J., Wang Y.H., Cui Y.D. (2014). Activation of PI3K/Akt pathway limits JNK-mediated apoptosis during EV71 infection. Virus Res..

[7] Wang H.Q., Hu J., Yan H.Y., Wu S., Li Y.H. (2017). Corydaline inhibits enterovirus 71 replication by regulating COX-2 expression. J. Asian Nat. Prod. Res..

[8] Wang Y.X., Fu H.G., Li Y.H., Jiang J.D., Song D.Q. (2012). Synthesis and biological evaluation of 8-substituted berberine derivatives as novel anti-mycobacterial agents. Acta Pharm. Sin. B.

[9] Wang Y.X., Li Y.H., Li Y.H., Gao R.M., Wang H.Q., Liu Y.X., Gao L.M., Lu Q.N., Jiang J.D., Song D.Q. (2012). Synthesis, structure-activity relationship and in vitro biological evaluation of N-arylethyl isoquinoline derivatives as Coxsackievirus B3 inhibitors. Bioorg. Med. Chem..

[10] Wang Y.X., Liu L., Zeng Q.X., Fan T.Y., Jiang J.D., Deng H.B., Song D.Q. (2017). Synthesis and identification of novel Berberine derivatives as potent inhibitors against TNF-α-induced NF-κB activation. Molecules.

[11] Wang Y.X., Pang W.Q., Zeng Q.X., Deng Z.S., Fan T.Y., Jiang J.D., Deng H.B., Song D.Q. (2018). Synthesis and biological evaluation of new berberine derivatives as cancer immunotherapy agents through targeting IDO1. Eur. J. Med. Chem..

[12] Liu Y.X., Xiao C.L., Wang Y.X., Li Y.H., Yang Y.H., Li Y.B., Bi C.W., Gao L.M., Jiang J.D., Song D.Q. (2012). Synthesis, structure-activity relationship and in vitro anti-mycobacterial evaluation of 13-n-octylberberine derivatives. Eur. J. Med. Chem..

[13] Li Y.H., Li Y., Yang P., Kong W.J., You X.F., Ren G., Deng H.B., Wang Y.M., Wang Y.X., Jiang J.D. (2010). Design, synthesis, and cholesterol-lowering efficacy for prodrugs of berberrubine. Bioorg. Med. Chem..

[14] Li Z.C., Kong X.B., Mai W.P., Sun G.C., Zhao S.Z. (2015). Synthesis and antimicrobial activity of 9-o-substituted palmatine derivatives. Indian J. Pharm. Sci..

[15] Wang B., Zhang H., Zhu M., Luo Z., Peng Y. (2012). MEK1-ERKs signal cascade is required for the replication of Enterovirus 71 (EV71). Antivir. Res..

[16] Wang C.Y., Wang P., Chen X.Q., Wang W., Jin Y. (2015). Saururus chinensis (Lour.) Baill blocks enterovirus 71 infection by hijacking MEK1-ERK signaling pathway. Antivir. Res..

[17] Wang C., Zhang H., Xu F.R., Niu Y., Wu Y., Wang X., Peng Y.H., Sun J., Liang L., Xu P. (2013). Substituted 3-benzylcoumarins as allosteric MEK1 inhibitors: Design, synthesis and biological evaluation as antiviral agents. Molecules.

[18] Tung W.H., Hsieh H.L., Lee I.T., Yang C.M. (2011). Enterovirus 71 modulates a COX-2/PGE2/cAMP- dependent viral replication in human neuroblastoma cells: Role of the c-Src/EGFR/p42/p44 MAPK/CREB signaling pathway. J. Cell. Biochem..

[19] Wang B., Ding L.X., Deng J., Zhang H., Zhu M., Yi T., Liu J., Xu P., Lu F.M., Peng Y.H. (2010). Replication of EV71 was suppressed by MEK1/2 inhibitor U0126. Chin. J. Biochem. Mol. Biol..

[20] Zhu M., Duan H., Gao M., Zhang H., Peng Y. (2015). Both ERK1 and ERK2 are required for enterovirus 71 (EV71) efficient replication. Viruses.

[21] Tung W.H., Hsieh H.L., Yang C.M. (2010). Enterovirus 71 induces COX-2 expression via MAPKs, NF-kappa B, and AP-1 in SK-N-SH cells: Role of PGE (2) in viral replication. Cell Signal..

[22] Shi W.F., Hou X.L., Peng H.J., Zhang L., Li Y.Y., Gu Z.W., Jiang Q.B., Shi M., Ji Y., Jiang J.T. (2014). MEK/ERK signaling pathway is required for enterovirus 71 replication in immature dendritic cells. Virol. J..

[23] Huang S.C., Chang C.L., Wang P.S., Sai Y.T., Liu H.S. (2009). Enterovirus 71-Induced autophagy detected in vitro and in vivo promotes viral replication. J. Med. Virol..

[24] Qin F., Zhang Z.B. (2015). Progress in studies on JNK signaling pathway and autophagy. J. Cent. South Univ..

[25] Feng F.B., Qiu H.Y. (2018). Effects of Artesunate on chondrocyte proliferation, apoptosis and autophagy through the PI3K/AKT/mTOR signaling pathway in rat models with rheumatoid arthritis. Biomed. Pharmacother..

