# Supplementary material for: Synthesis and Evolution of Berberine Derivatives as a New Class of Antiviral Agents against Enterovirus 71 through the MEK/ERK Pathway and Autophagy
Source: Molecules. 2018 Aug 20;23(8):2084. doi: 10.3390/molecules23082084 (PMC6222558; doi:10.3390/molecules23082084)
Supplement: Supplementary file 1 [file molecules-23-02084-s001.pdf]

Supporting Information

Synthesis and Evolution of Berberine Derivatives as a New Class of Antiviral Agents against Enterovirus 71 through MEK/ERK Pathway and Autophagy

Yan–Xiang Wang, Lu Yang, Hui–Qiang Wang, Xiao–Qiang Zhao, Ting Liu, Ying–Hong Li, Qing–Xuan Zeng, Yu–Huan Li, Dan–Qing Song

1. ^{1}H NMR, ^{13}C NMR, HRMS-ESI spectra

2a

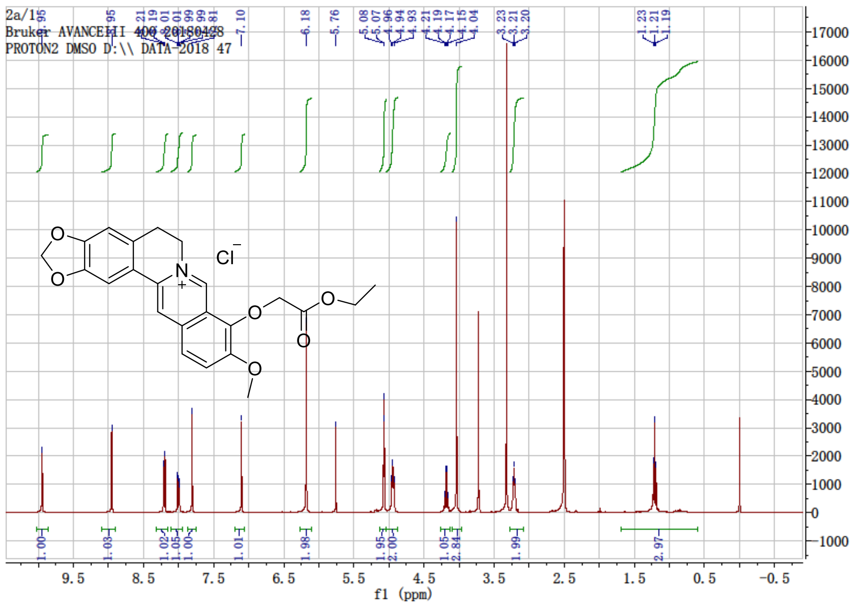

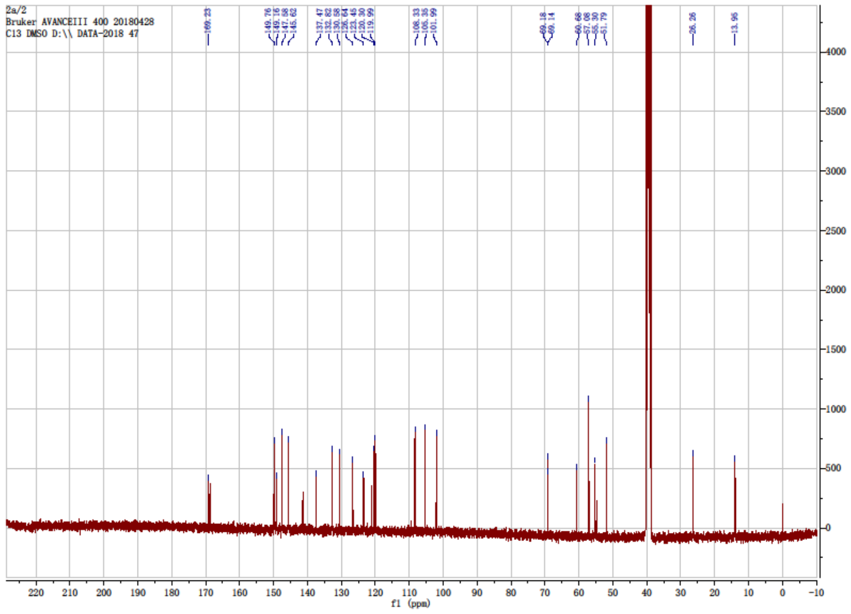

E:\HRMS\2018\04\27\51_180427104716 4/27/2018 11:20:09 AM 51

51_180427104716 #57 RT: 0.46 AV: 1 NL: 5.34E8
T: FTMS + c ESI Full ms [100.00-1100.00]

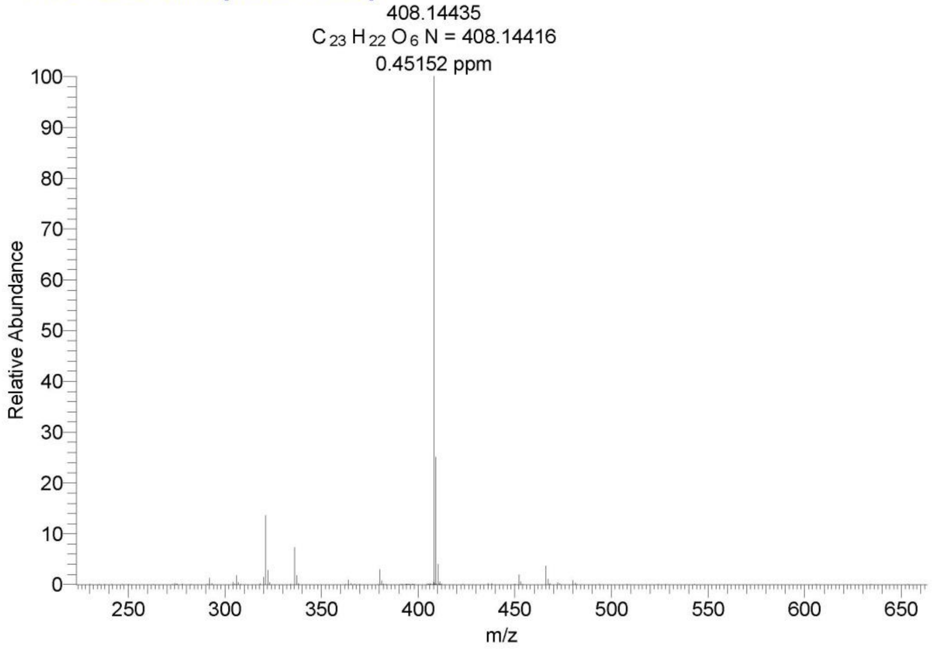

2b

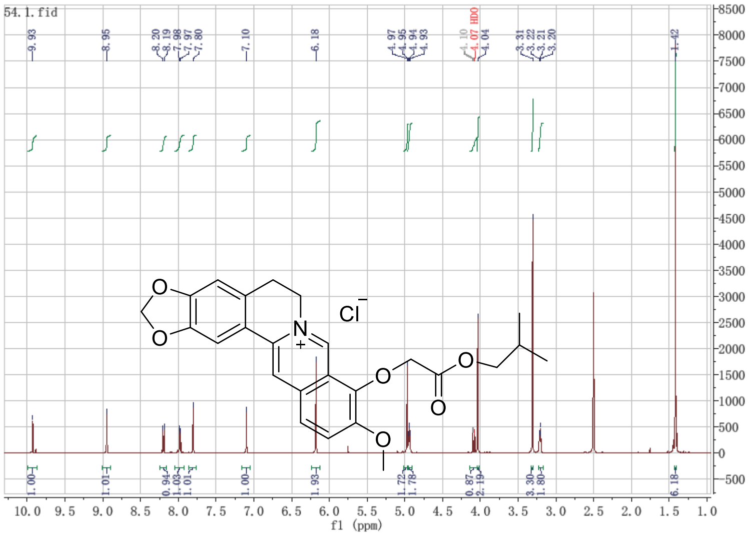

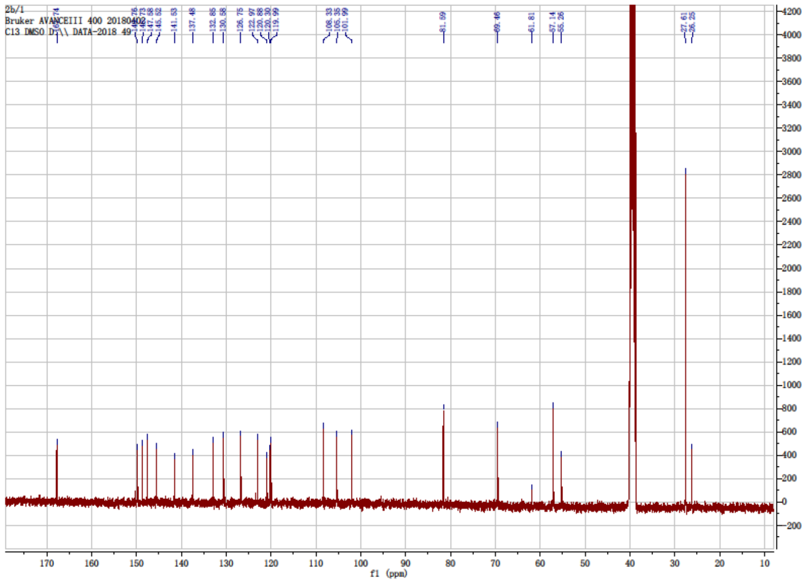

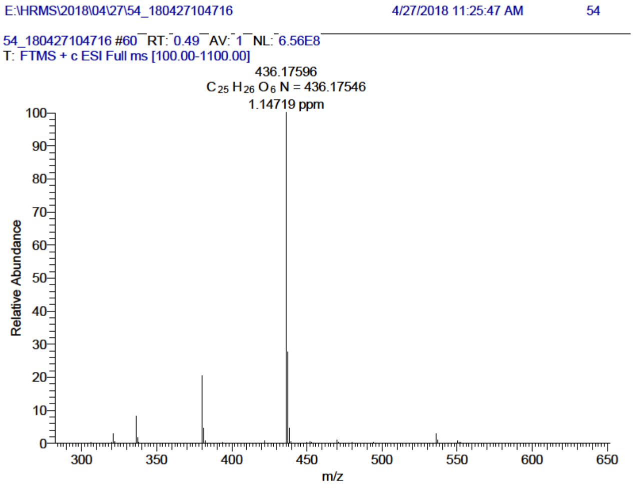
E:\HRMS\2018\04\27\54_180427104716
4/27/2018 11:25:47 AM
54
54_180427104716 #60 RT: 0.49 AV: 1 NL: 6.56E8
T: FTMS + c ESI Full ms [100.00-1100.00]
436.17596
C25 H26 O6 N = 436.17546
1.14719 ppm
Relative Abundance
m/z

2c

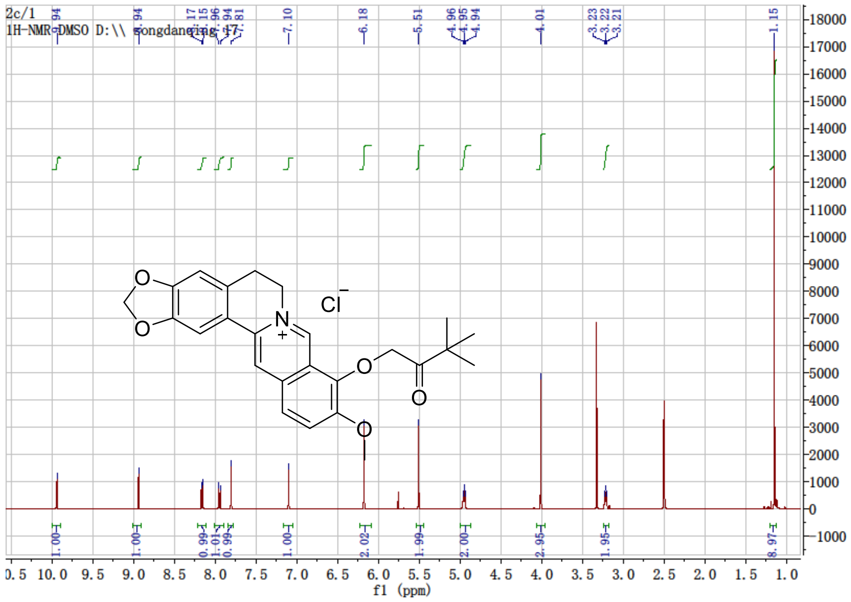

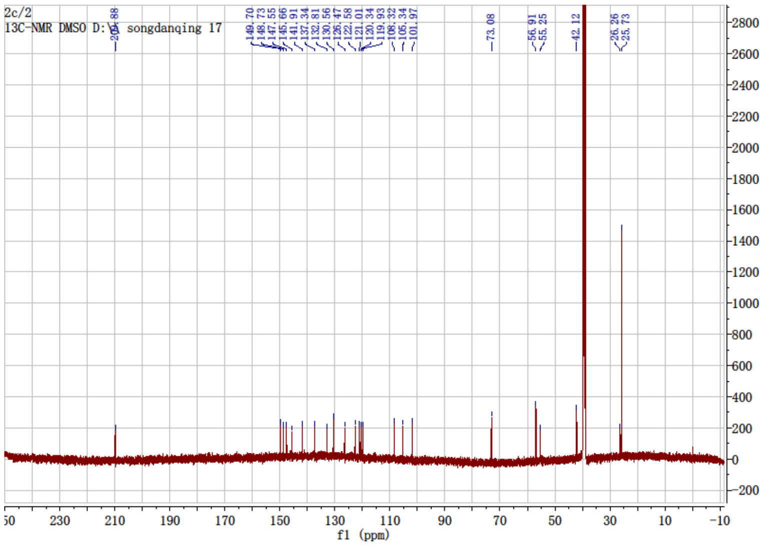

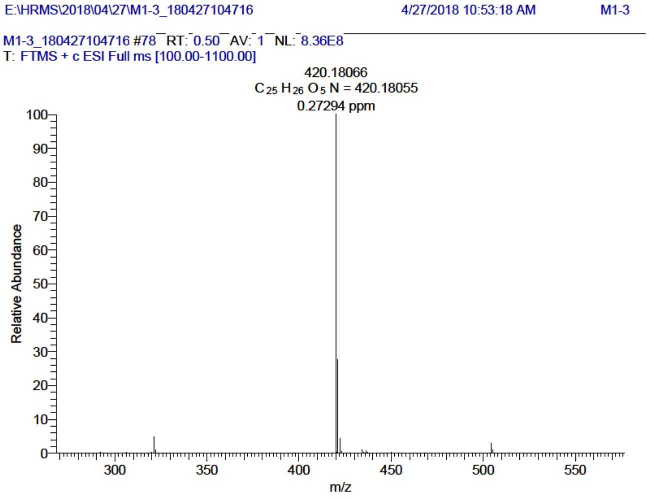
E:\HRMS\2018\04\27\M1-3_180427104716
4/27/2018 10:53:18 AM
M1-3
M1-3_180427104716 #78 RT: 0.50 AV: 1 NL: 8.36E8
T: FTMS + c ESI Full ms [100.00-1100.00]
420.18066
C25 H26 O5 N = 420.18055
0.27294 ppm
Relative Abundance
m/z

2d

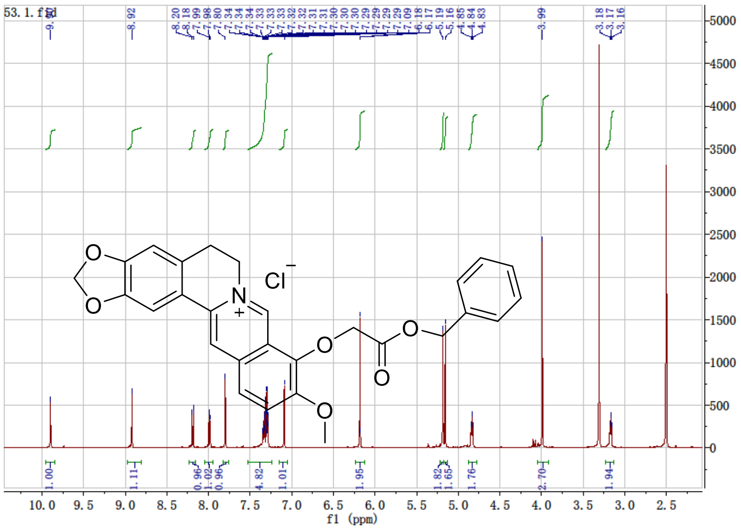

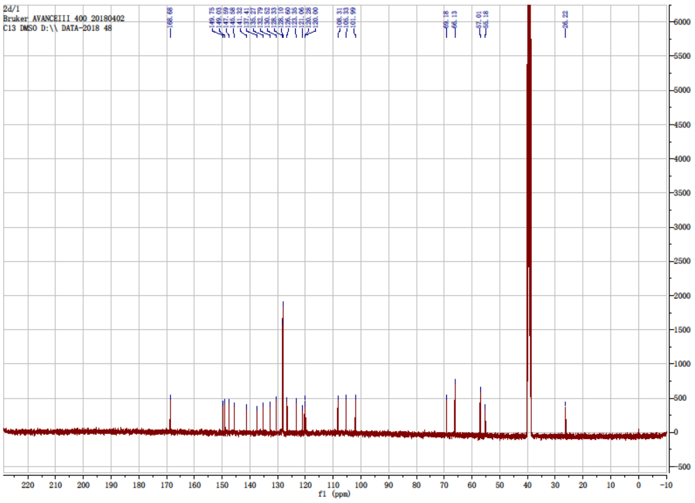

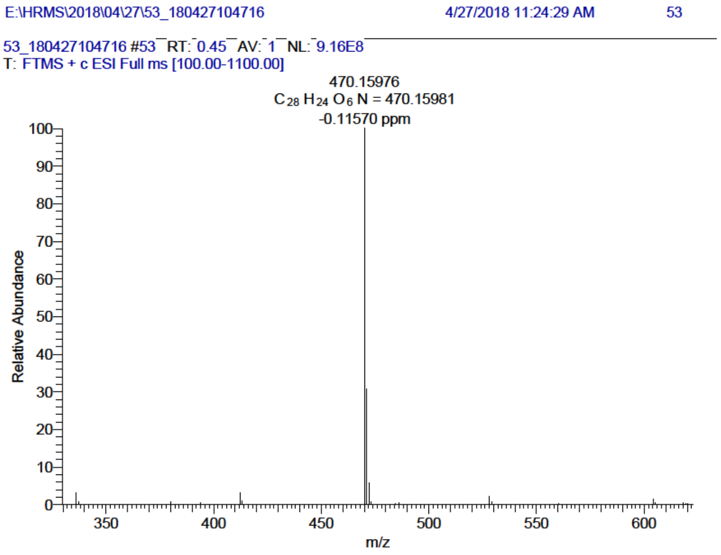
E:\HRMS\2018\04\27\53_180427104716
4/27/2018 11:24:29 AM
53
53_180427104716 #53 RT: 0.45 AV: 1 NL: 9.16E8
T: FTMS + c ESI Full ms [100.00-1100.00]
470.15976
C28 H24 O6 N = 470.15981
-0.11570 ppm
Relative Abundance
m/z

2d-a

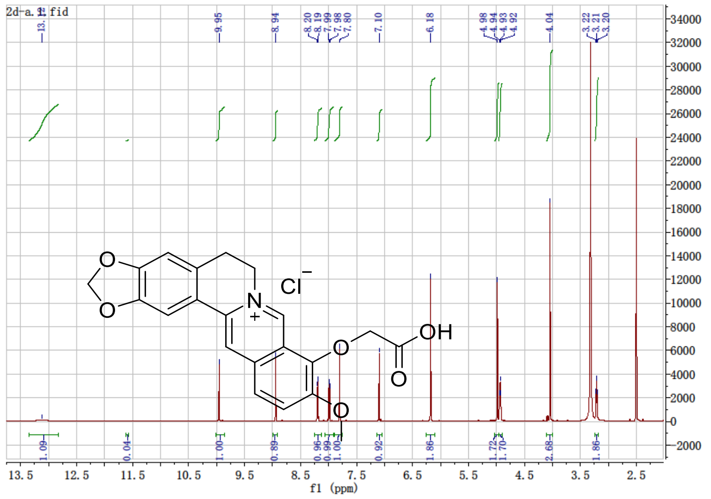

2d-a.1.fid
f1 (ppm)

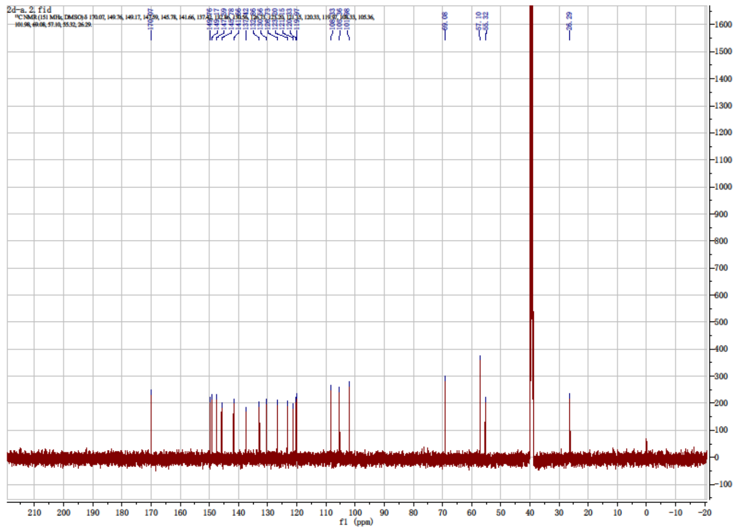

2d-a.2.fid
f1 (ppm)

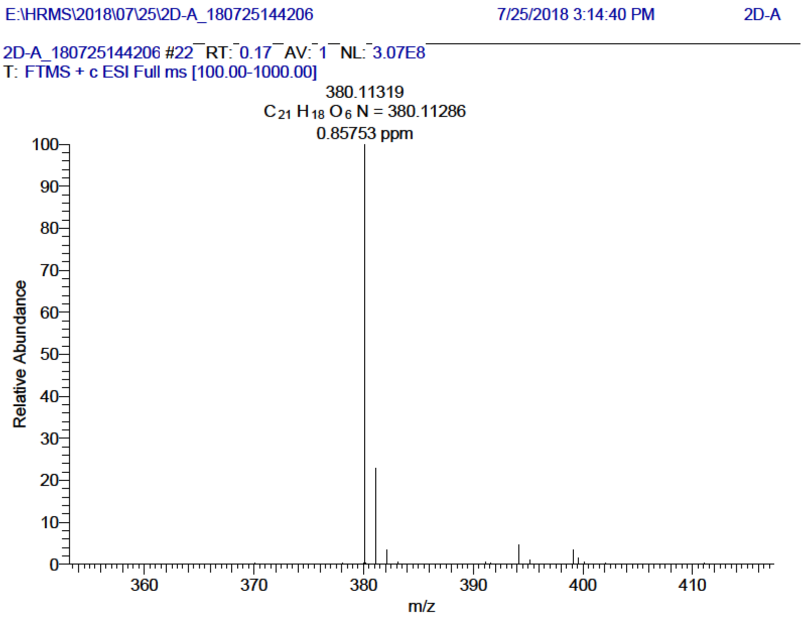

E:\HRMS\2018\07\25\2D-A_180725144206
7/25/2018 3:14:40 PM
2D-A
2D-A_180725144206 #22 RT: 0.17 AV: 1 NL: 3.07E8
T: FTMS + c ESI Full ms [100.00-1000.00]
380.11319
C21 H18 O6 N = 380.11286
0.85753 ppm
Relative Abundance
m/z

2e

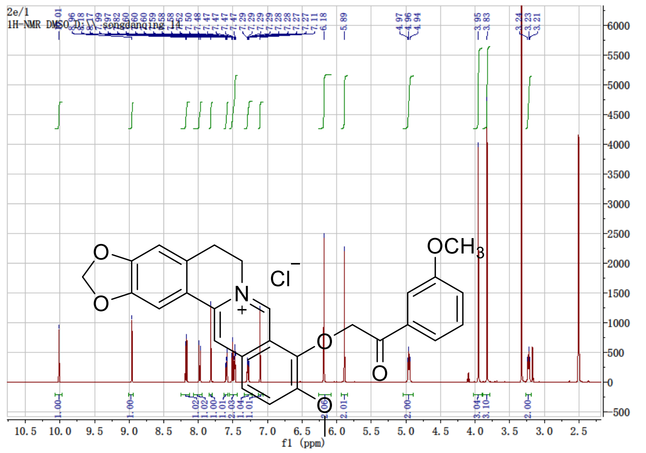
2e/1
OCH3
Cl
f1 (ppm)

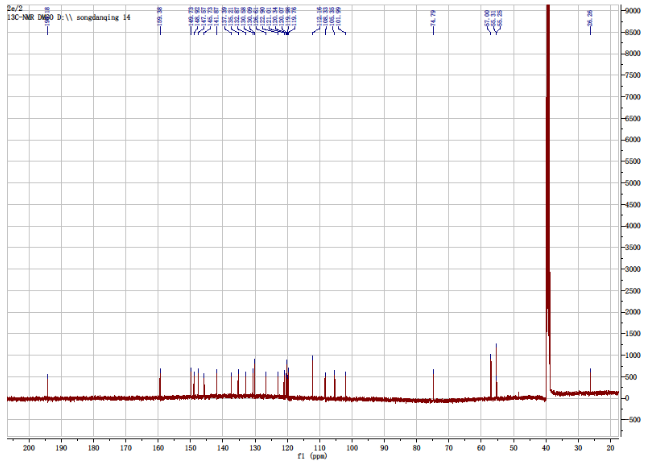
2e/2
f1 (ppm)

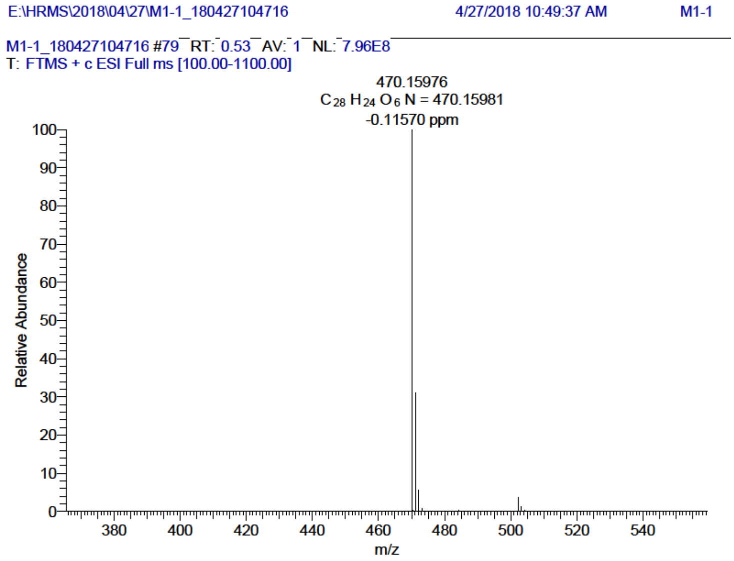
E:\HRMS\2018\04\27\M1-1_180427104716
4/27/2018 10:49:37 AM
M1-1
M1-1_180427104716 #79 RT: 0.53 AV: 1 NL: 7.96E8
T: FTMS + c ESI Full ms [100.00-1100.00]
470.15976
C28 H24 O6 N = 470.15981
-0.11570 ppm
Relative Abundance
m/z

2f

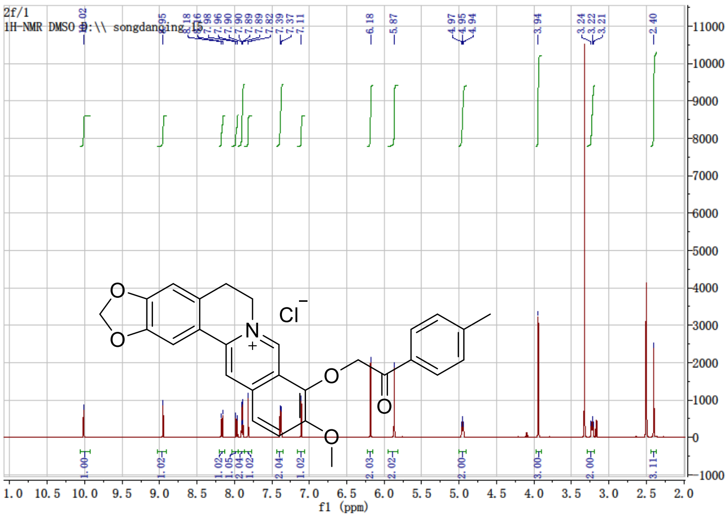

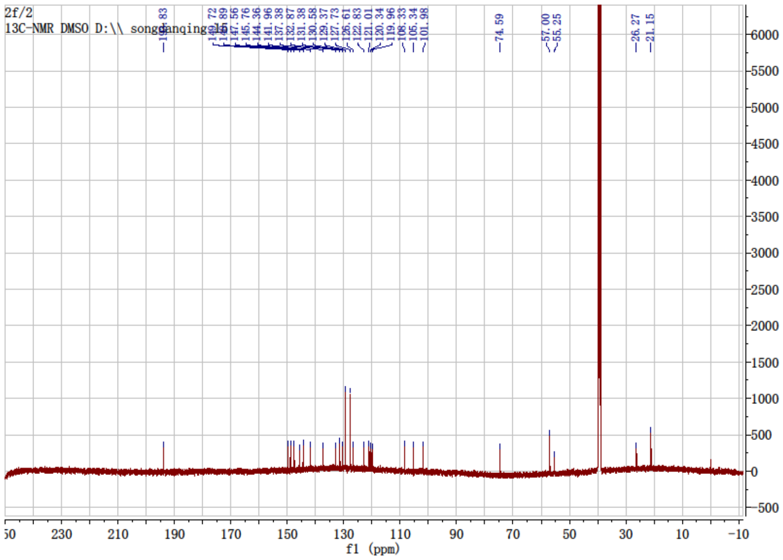

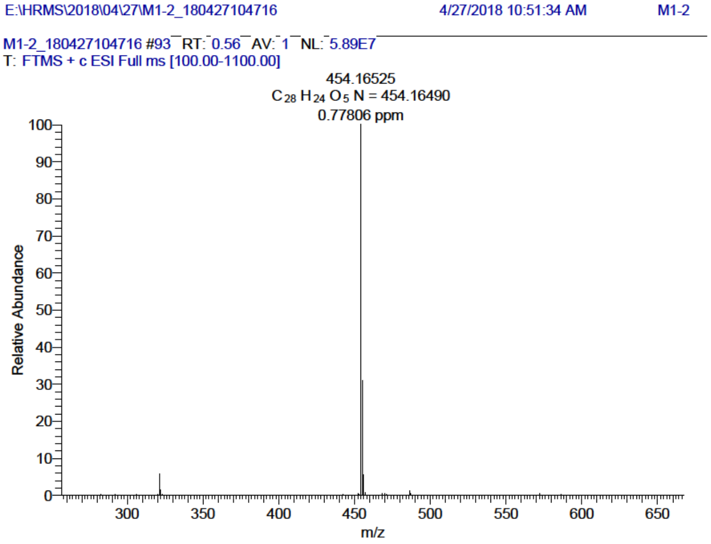
E:\HRMS\2018\04\27\M1-2_180427104716
4/27/2018 10:51:34 AM
M1-2
M1-2_180427104716 #93 RT: 0.56 AV: 1 NL: 5.89E7
T: FTMS + c ESI Full ms [100.00-1100.00]
454.16525
C28 H24 O5 N = 454.16490
0.77806 ppm
Relative Abundance
m/z

2g

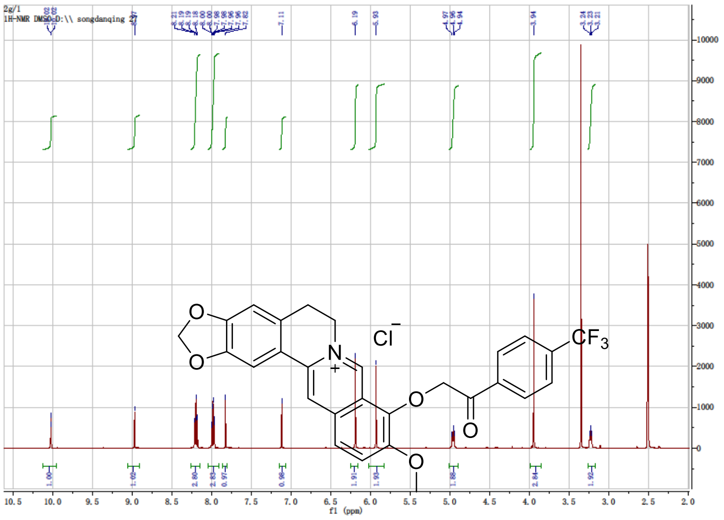

2g/1
1H-NMR DMSO-D:\\ songdanqing 27
Cl
CF3
f1 (ppm)

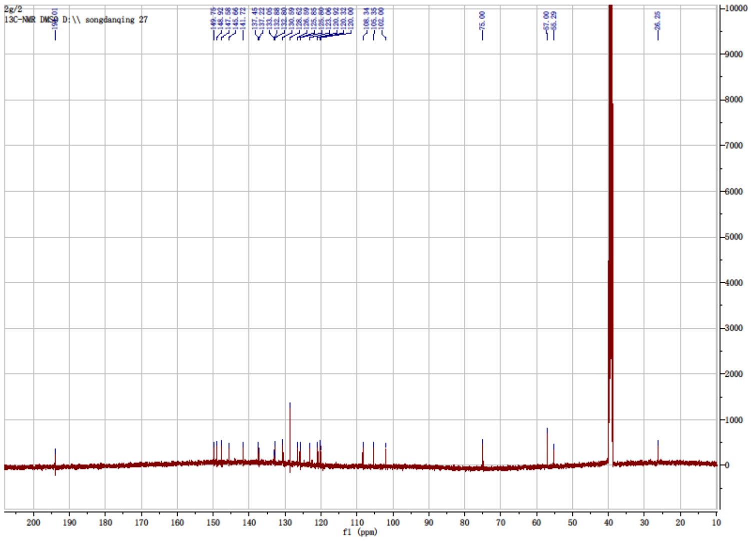

2g/2
13C-NMR DMSO D:\\ songdanqing 27
f1 (ppm)

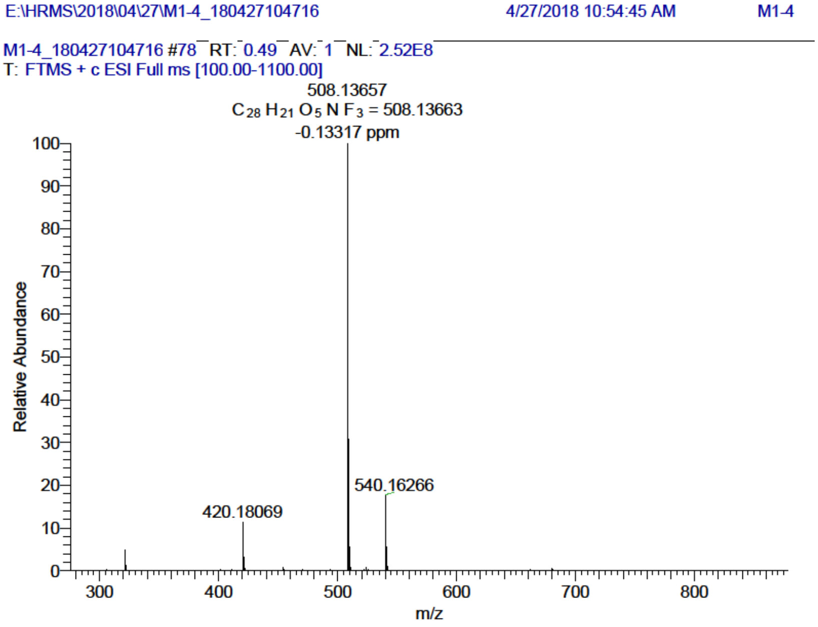

E:\HRMS\2018\04\27\M1-4_180427104716
4/27/2018 10:54:45 AM
M1-4
M1-4_180427104716 #78 RT: 0.49 AV: 1 NL: 2.52E8
T: FTMS + c ESI Full ms [100.00-1100.00]
508.13657
C28 H21 O5 N F3 = 508.13663
-0.13317 ppm
540.16266
420.18069
Relative Abundance
m/z

2h

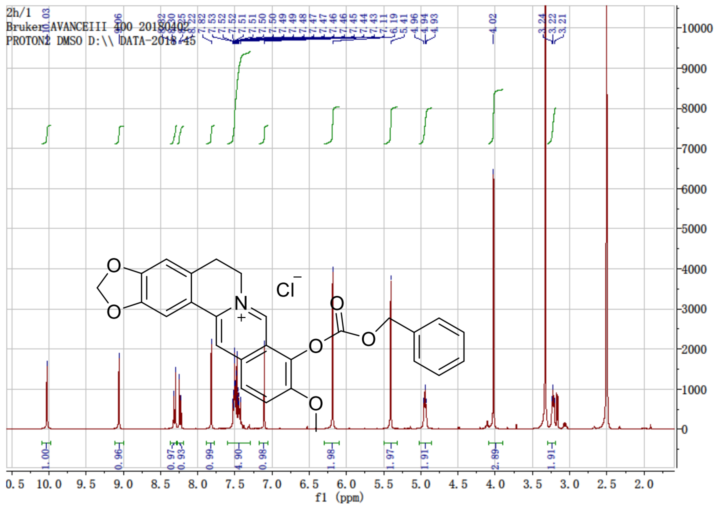
2h/1
Bruker AVANCEIII 400 20180402
PROTON2 DMSO D:\\ DATA-2018 45
Cl⁻
f1 (ppm)

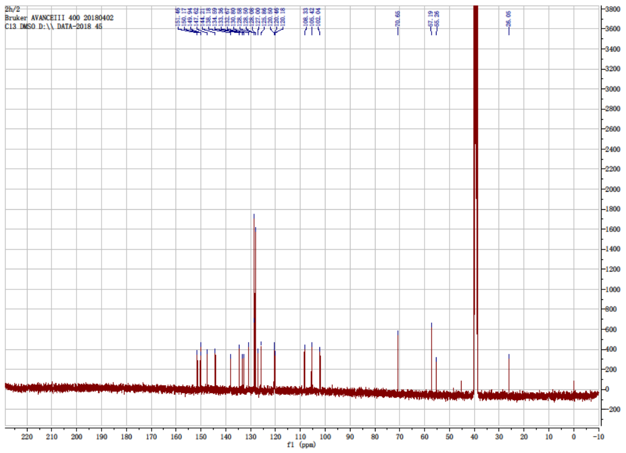
2h/2
Bruker AVANCEIII 400 20180402
C13 DMSO D:\\ DATA-2018 45
f1 (ppm)

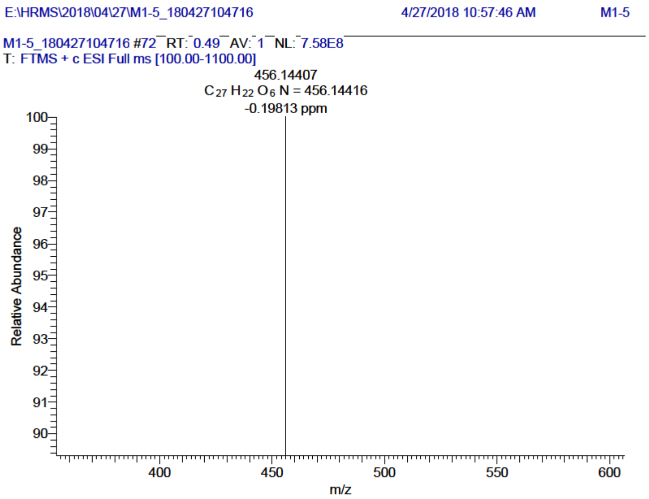
E:\HRMS\2018\04\27\M1-5_180427104716
4/27/2018 10:57:46 AM
M1-5
M1-5_180427104716 #72 RT: 0.49 AV: 1 NL: 7.58E8
T: FTMS + c ESI Full ms [100.00-1100.00]
456.14407
C27 H22 O6 N = 456.14416
-0.19813 ppm
Relative Abundance
m/z

2i

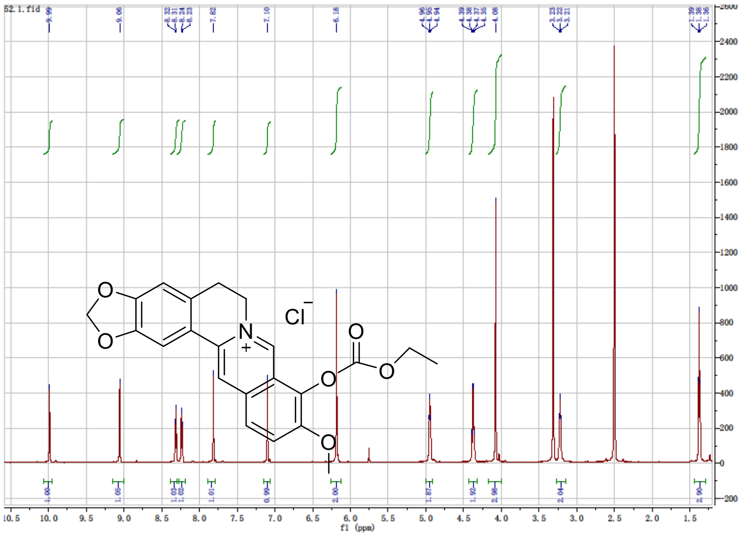

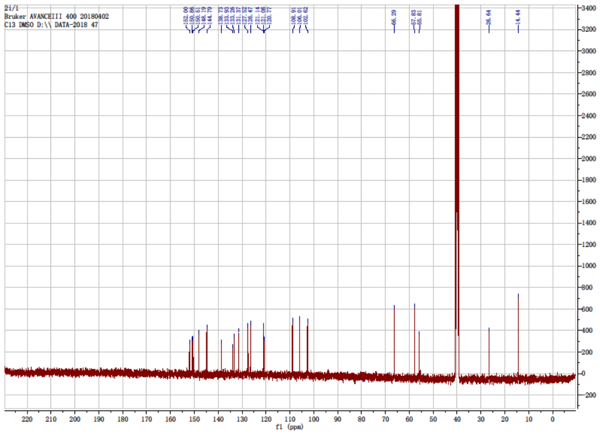

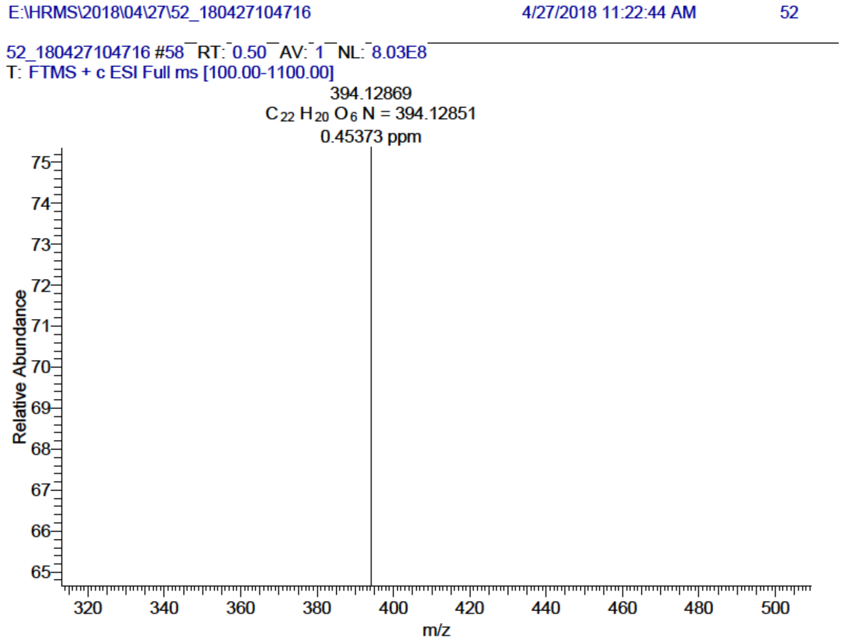

E:\HRMS\2018\04\27\52_180427104716
4/27/2018 11:22:44 AM
52
52_180427104716 #58 RT: 0.50 AV: 1 NL: 8.03E8
T: FTMS + c ESI Full ms [100.00-1100.00]
394.12869
C22 H20 O6 N = 394.12851
0.45373 ppm
Relative Abundance
m/z

4a

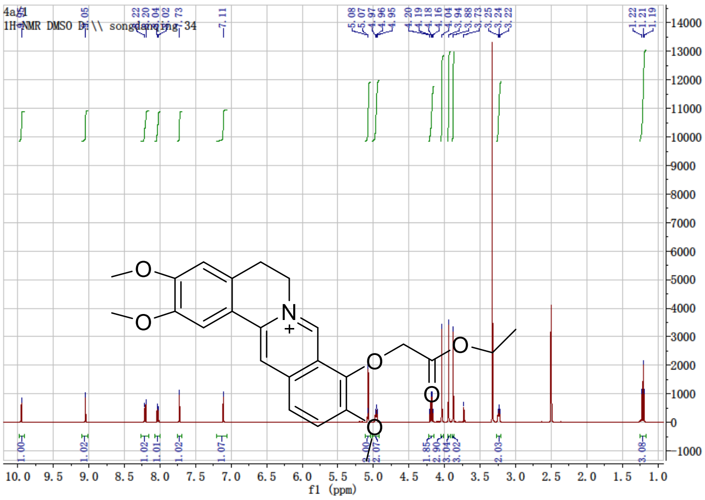
4a/1
1H-NMR DMSO D:\\ songdanqing 34
f1 (ppm)

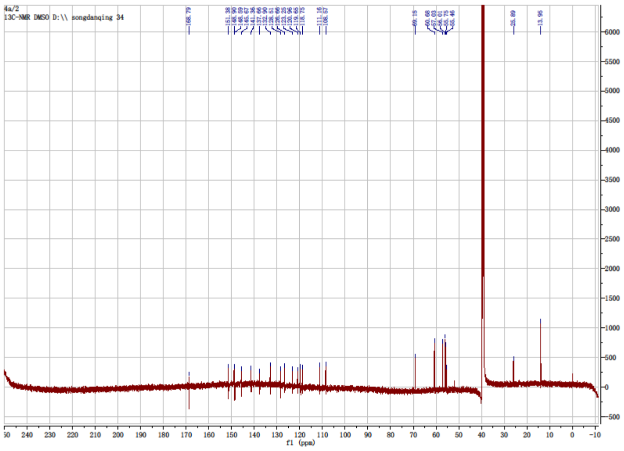
4a/2
13C-NMR DMSO D:\\ songdanqing 34
f1 (ppm)

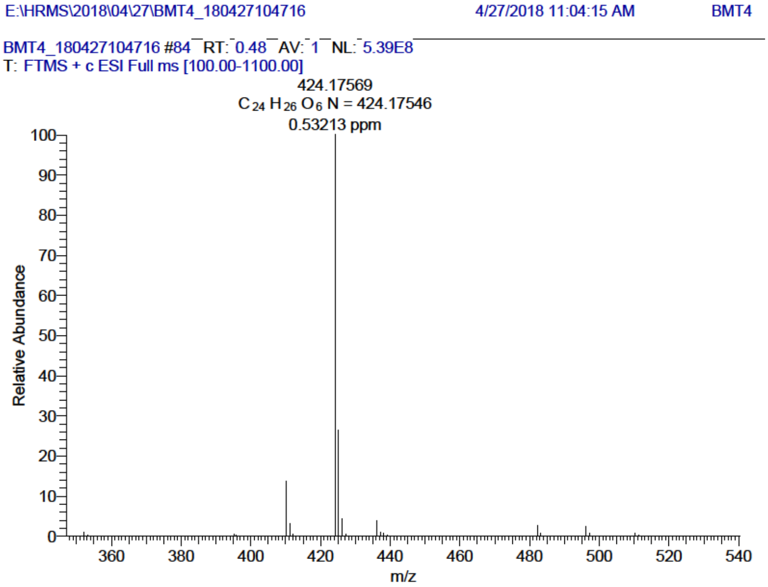
E:\HRMS\2018\04\27\BMT4_180427104716
4/27/2018 11:04:15 AM
BMT4
BMT4_180427104716 #84 RT: 0.48 AV: 1 NL: 5.39E8
T: FTMS + c ESI Full ms [100.00-1100.00]
424.17569
C 24 H 26 O 6 N = 424.17546
0.53213 ppm
Relative Abundance
m/z

4b

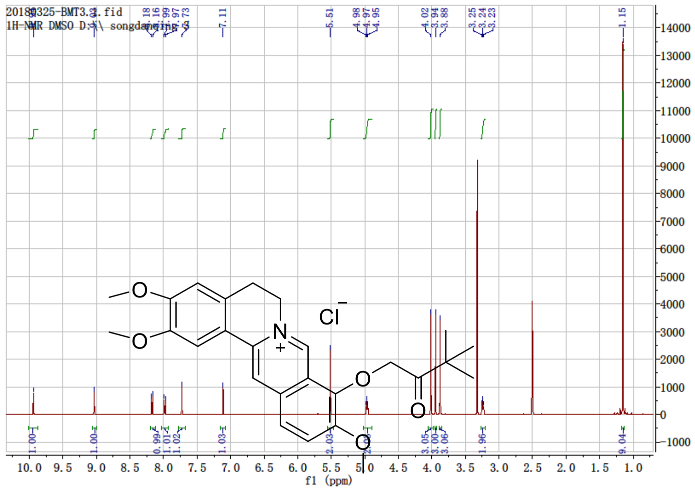

20180325-BMT3.1.fid
1H-NMR DMSO D:\\ songdanqing 3
Cl⁻
f1 (ppm)

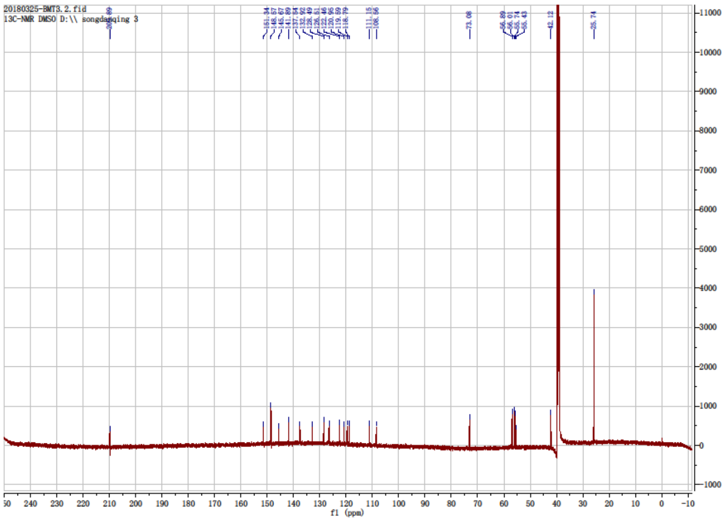

20180325-BMT3.2.fid
13C-NMR DMSO D:\\ songdanqing 3
f1 (ppm)

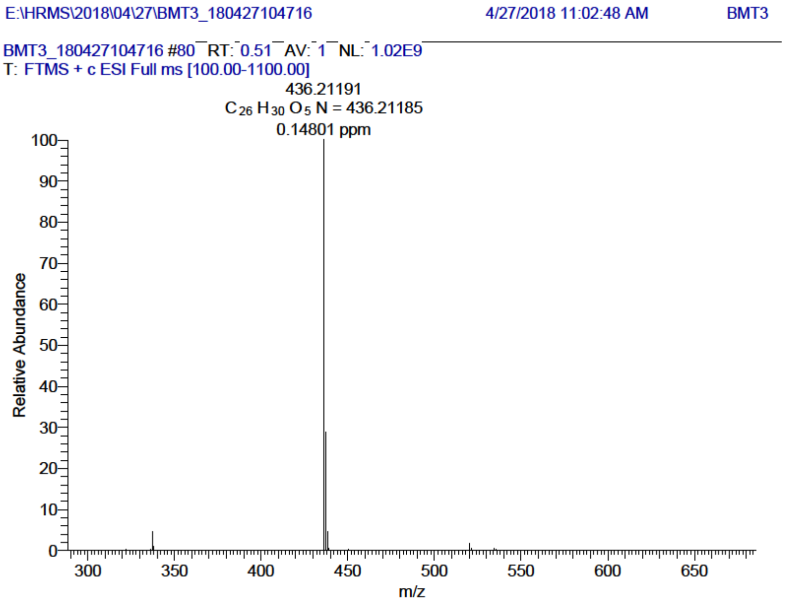

E:\HRMS\2018\04\27\BMT3_180427104716
4/27/2018 11:02:48 AM
BMT3
BMT3_180427104716 #80 RT: 0.51 AV: 1 NL: 1.02E9
T: FTMS + c ESI Full ms [100.00-1100.00]
436.21191
C26 H30 O5 N = 436.21185
0.14801 ppm
Relative Abundance
m/z

4c

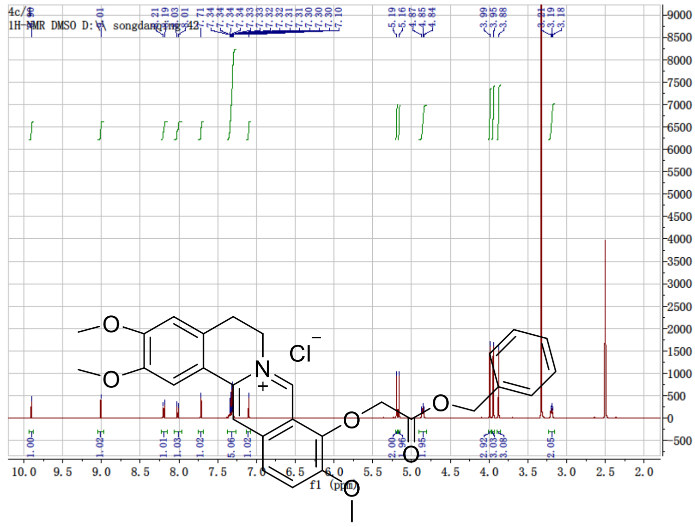

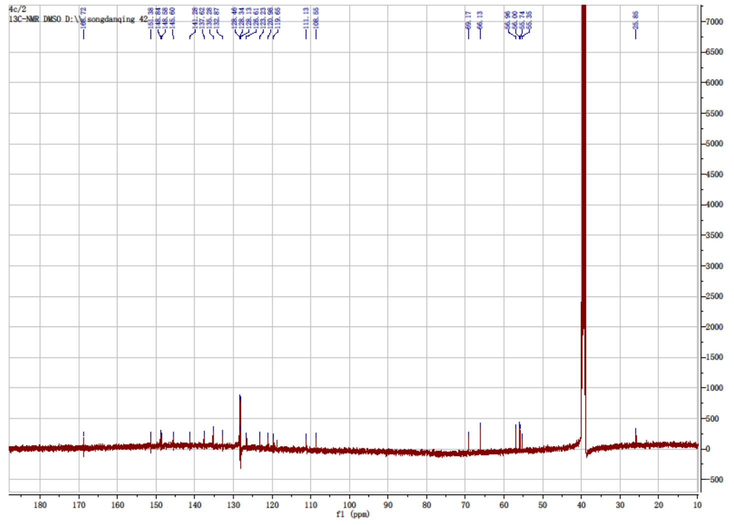

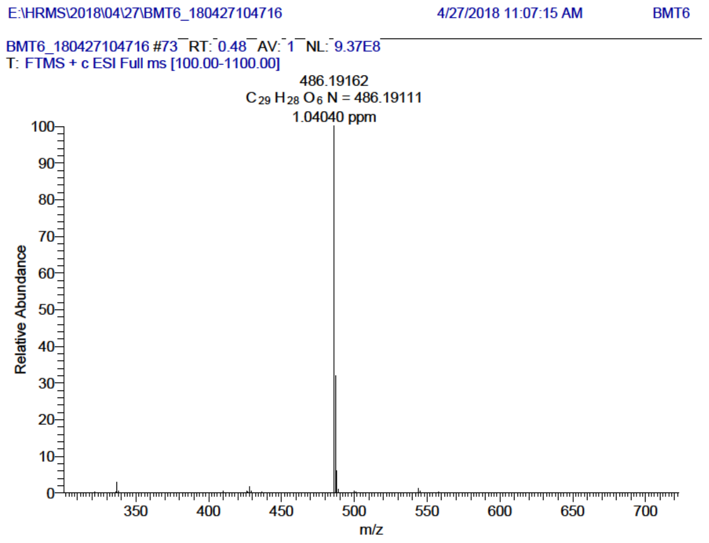

4d

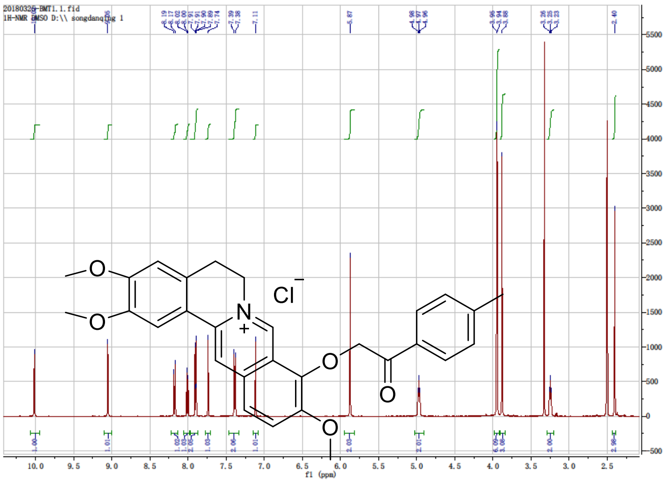
20180325-BMT1.1.fid
1H-NMR DMSO D:\\ songdanqing 1
f1 (ppm)
Cl
N
O
O

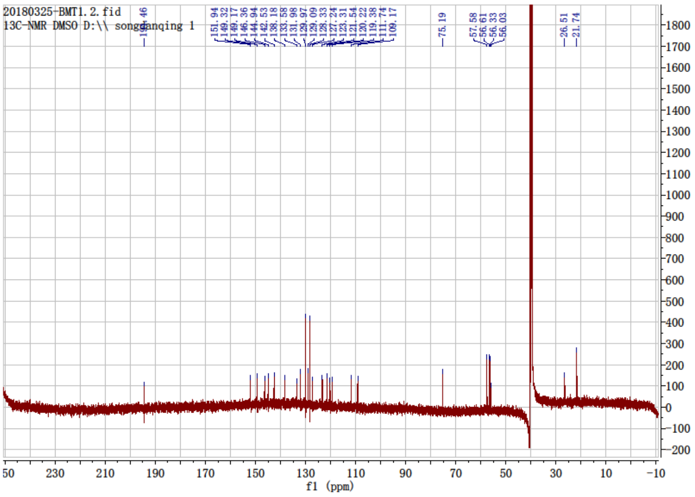
20180325-BMT1.2.fid
13C-NMR DMSO D:\\ songdanqing 1
f1 (ppm)

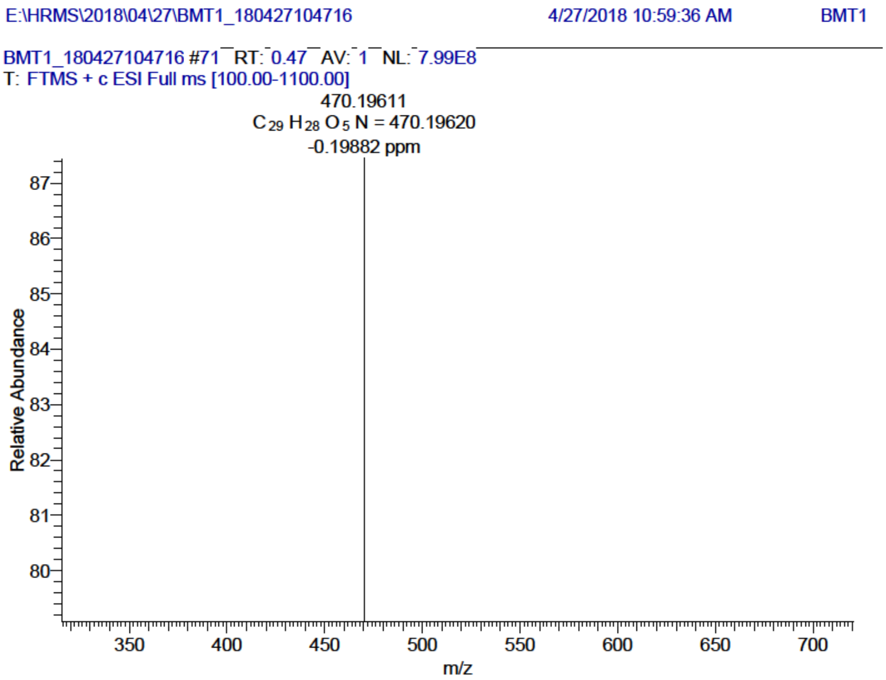
E:\HRMS\2018\04\27\BMT1_180427104716
4/27/2018 10:59:36 AM
BMT1
BMT1_180427104716 #71 RT: 0.47 AV: 1 NL: 7.99E8
T: FTMS + c ESI Full ms [100.00-1100.00]
470.19611
C29 H28 O5 N = 470.19620
-0.19882 ppm
Relative Abundance
m/z

4e

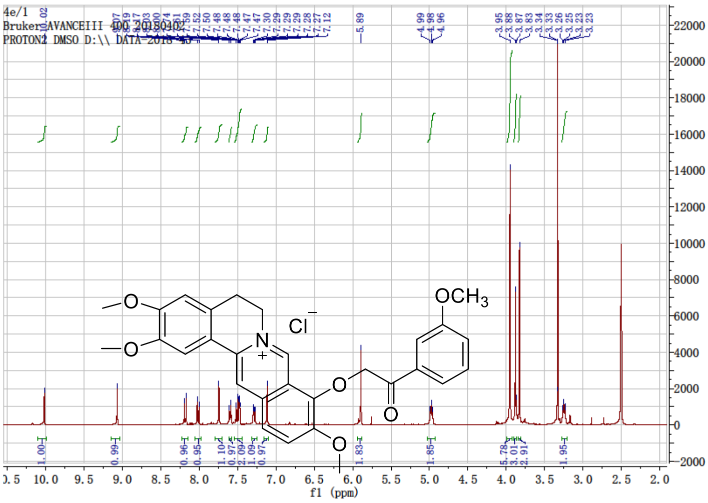

4e/1
OCH3
Cl
f1 (ppm)

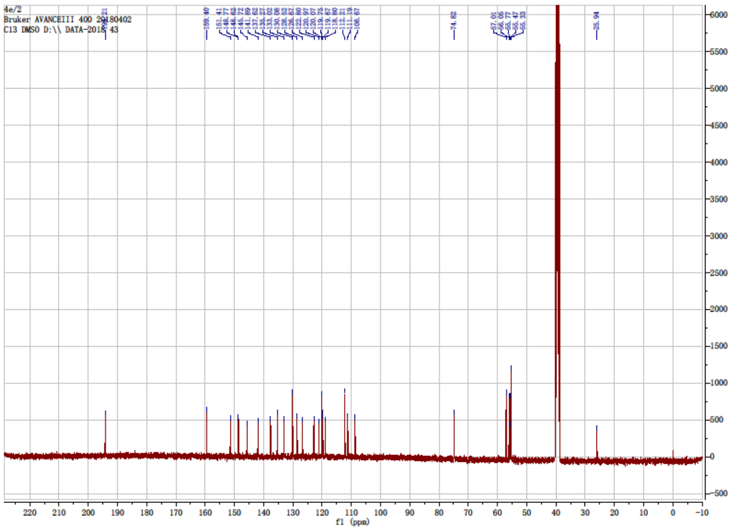

4e/2
f1 (ppm)

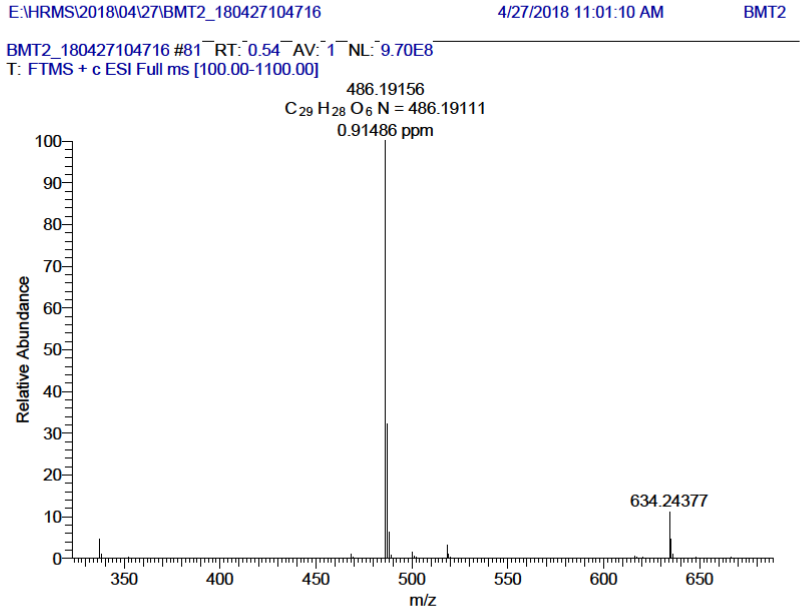

E:\HRMS\2018\04\27\BMT2_180427104716
4/27/2018 11:01:10 AM
BMT2
BMT2_180427104716 #81 RT: 0.54 AV: 1 NL: 9.70E8
T: FTMS + c ESI Full ms [100.00-1100.00]
486.19156
C 29 H 28 O 6 N = 486.19111
0.91486 ppm
634.24377
Relative Abundance
m/z

4f

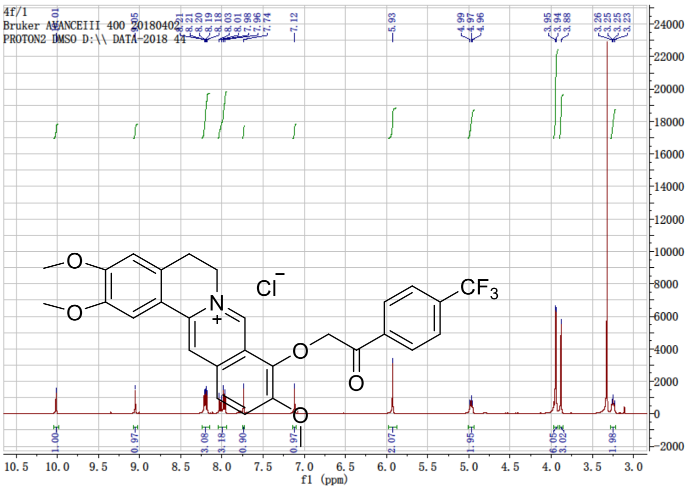

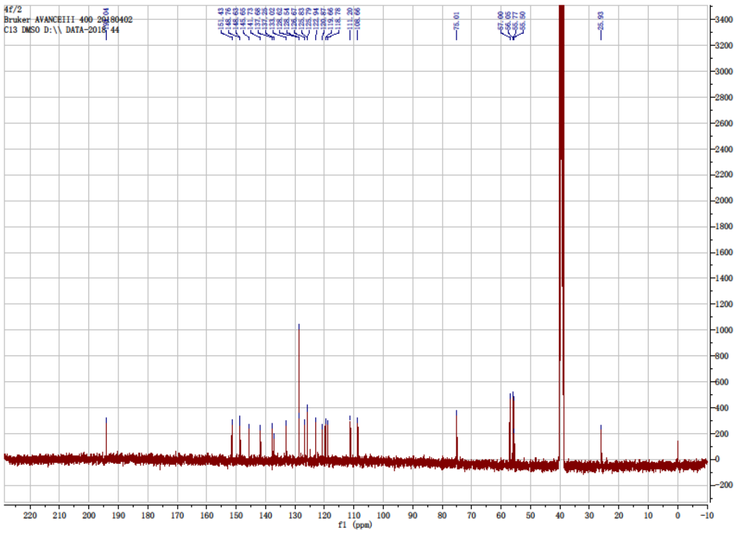

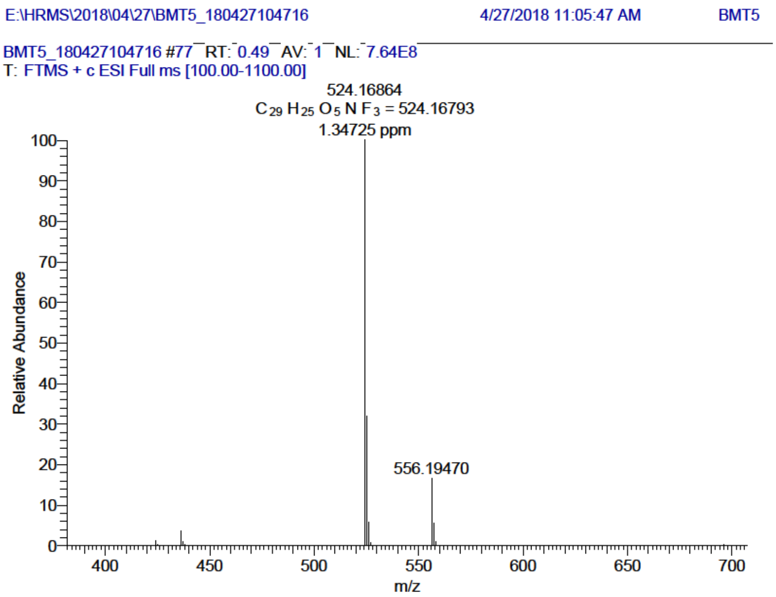
E:\HRMS\2018\04\27\BMT5_180427104716
4/27/2018 11:05:47 AM
BMT5
BMT5_180427104716 #77 RT: 0.49 AV: 1 NL: 7.64E8
T: FTMS + c ESI Full ms [100.00-1100.00]
524.16864
C29 H25 O5 N F3 = 524.16793
1.34725 ppm
556.19470
Relative Abundance
m/z

4g

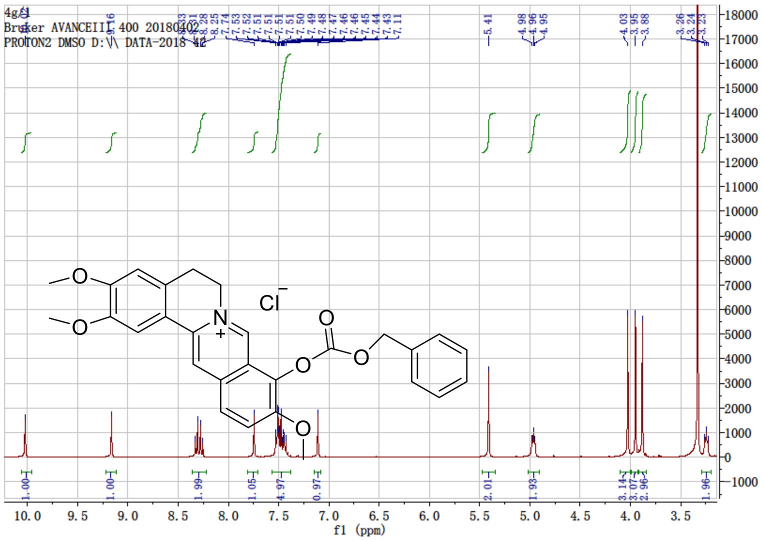

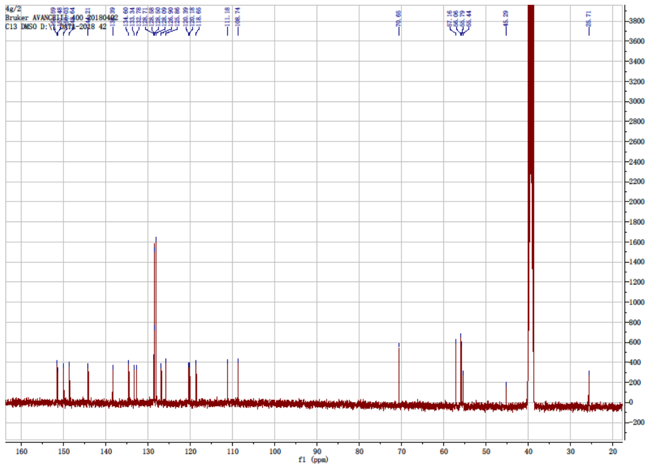

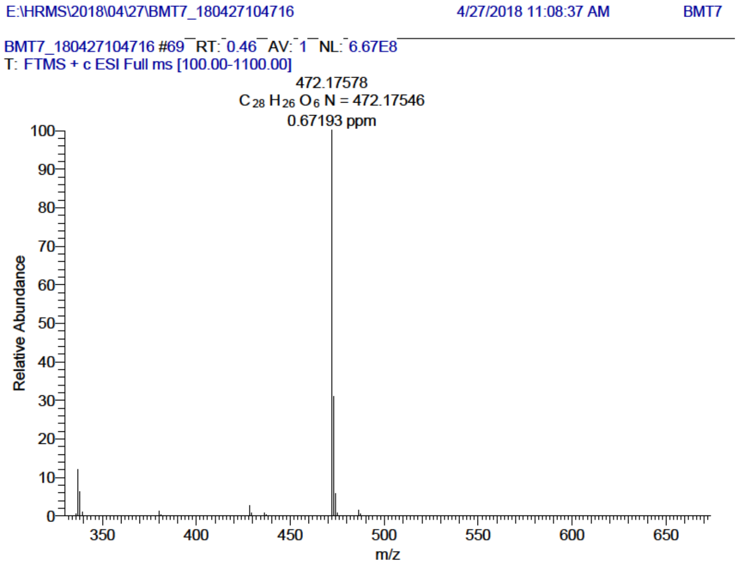
E:\HRMS\2018\04\27\BMT7_180427104716
4/27/2018 11:08:37 AM
BMT7
BMT7_180427104716 #69 RT: 0.46 AV: 1 NL: 6.67E8
T: FTMS + c ESI Full ms [100.00-1100.00]
472.17578
C 28 H 26 O 6 N = 472.17546
0.67193 ppm
Relative Abundance
m/z

5a

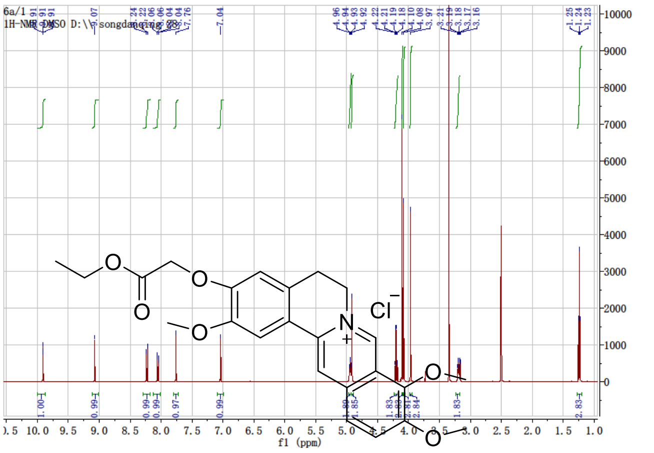

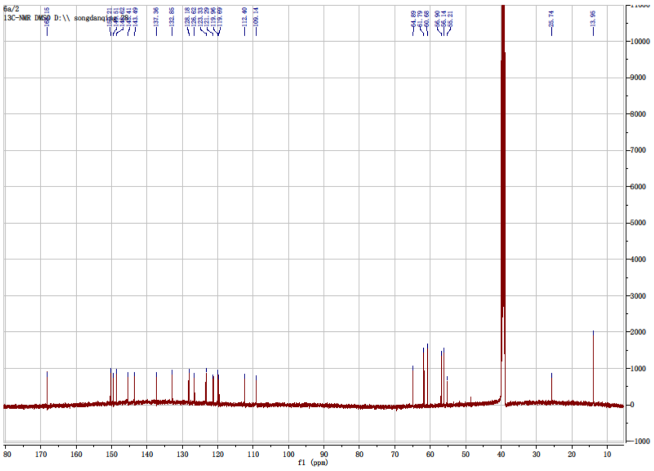

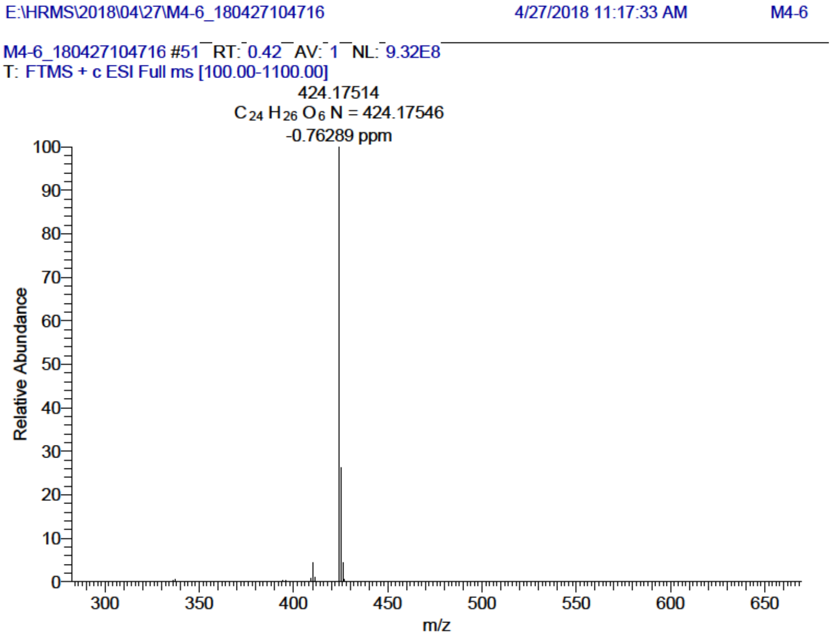
E:\HRMS\2018\04\27\M4-6_180427104716
4/27/2018 11:17:33 AM
M4-6
M4-6_180427104716 #51 RT: 0.42 AV: 1 NL: 9.32E8
T: FTMS + c ESI Full ms [100.00-1100.00]
424.17514
C24 H26 O6 N = 424.17546
-0.76289 ppm
Relative Abundance
m/z

5b

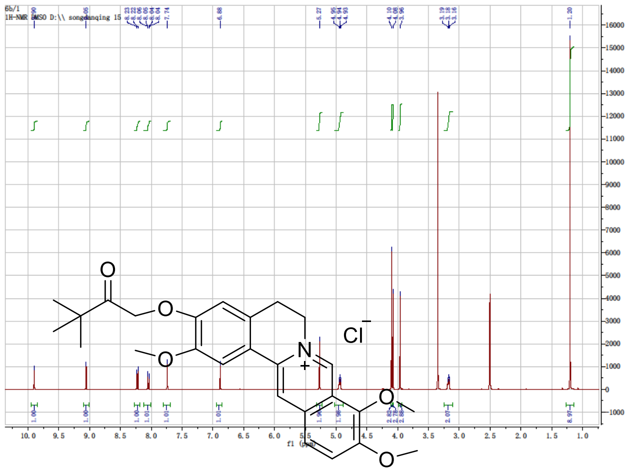
1H-NMR DMSO D:\\ songdanqing 15
Cl⁻
f1 (ppm)

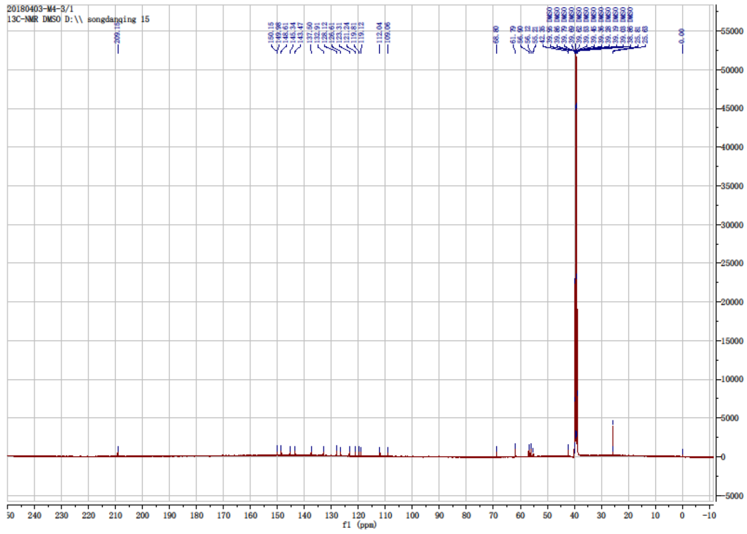
20180403-M4-3/1
13C-NMR DMSO D:\\ songdanqing 15
f1 (ppm)

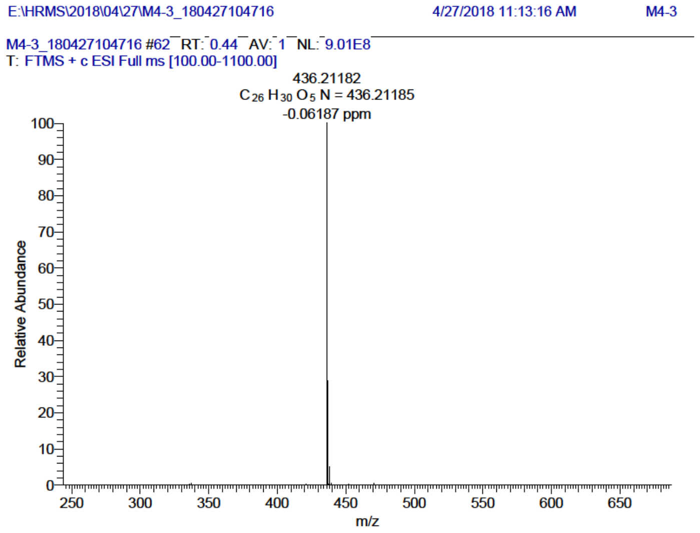
E:\HRMS\2018\04\27\M4-3_180427104716
4/27/2018 11:13:16 AM
M4-3
M4-3_180427104716 #62 RT: 0.44 AV: 1 NL: 9.01E8
T: FTMS + c ESI Full ms [100.00-1100.00]
436.21182
C26 H30 O5 N = 436.21185
-0.06187 ppm
Relative Abundance
m/z

5c

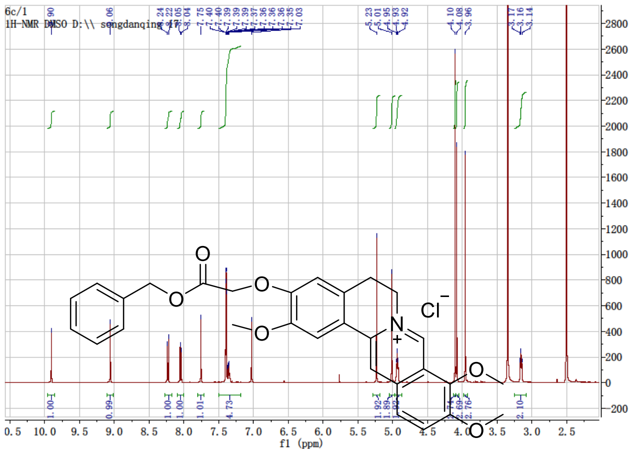

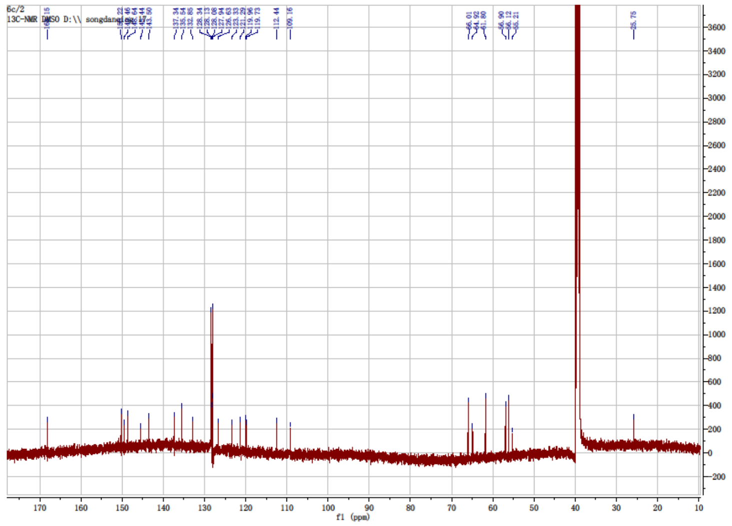

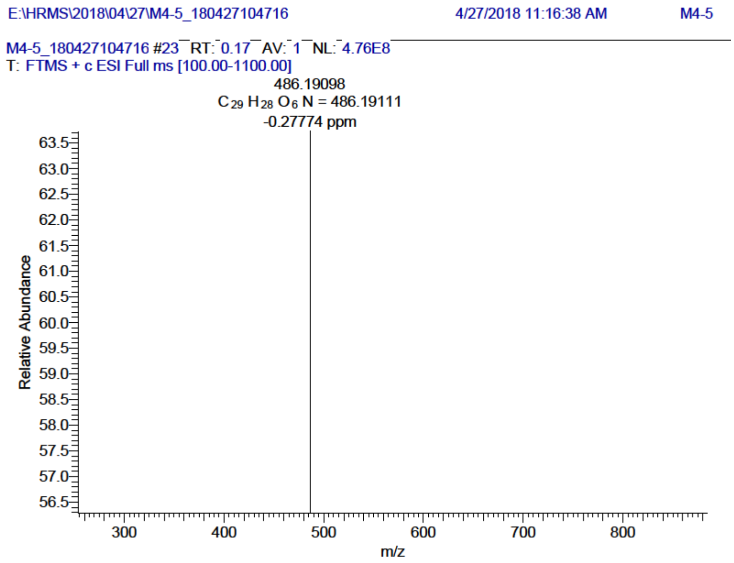

5d

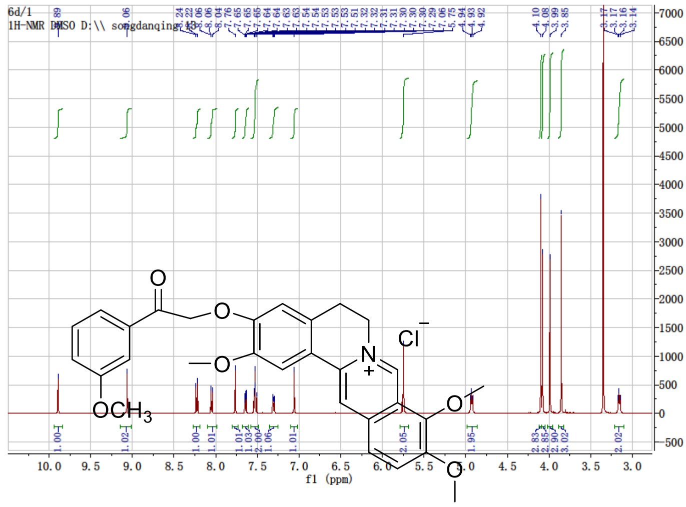
6d/1
1H-NMR DMSO D:\\ songdanqing\43
Cl
N
O
OCH3
f1 (ppm)

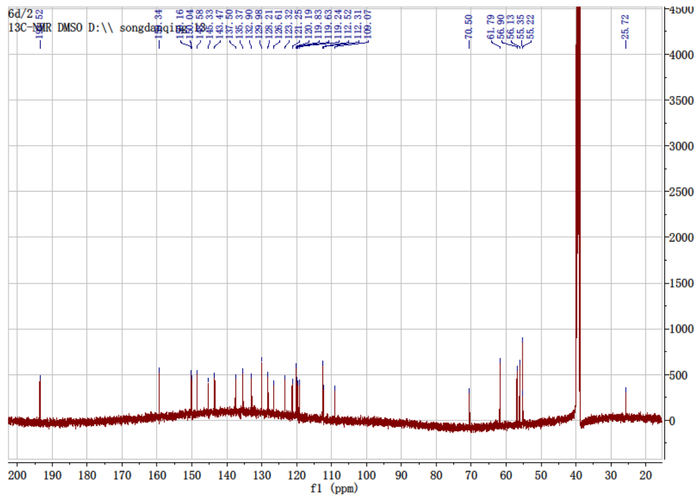
6d/2
13C-NMR DMSO
f1 (ppm)

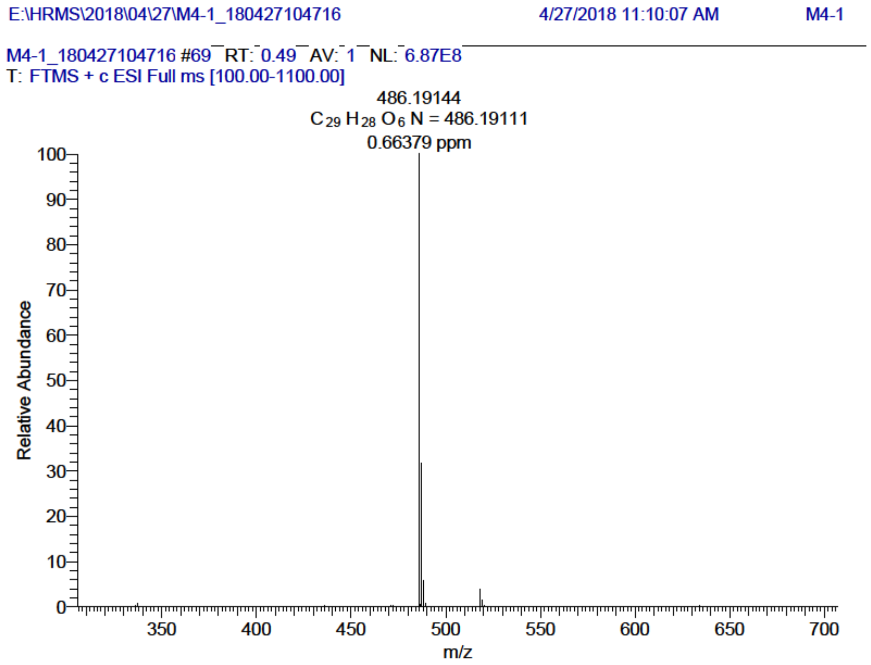
E:\HRMS\2018\04\27\M4-1_180427104716
4/27/2018 11:10:07 AM
M4-1
M4-1_180427104716 #69 RT: 0.49 AV: 1 NL: 6.87E8
T: FTMS + c ESI Full ms [100.00-1100.00]
486.19144
C29 H28 O6 N = 486.19111
0.66379 ppm
Relative Abundance
m/z

5e

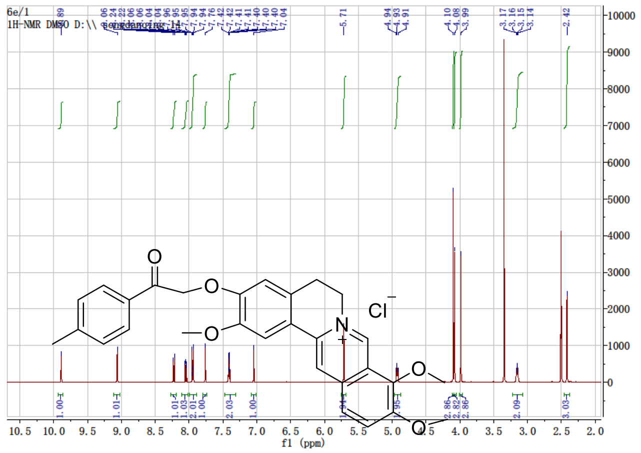
6e/1
1H-NMR DMSO D:\\ songdanqing 14
f1 (ppm)

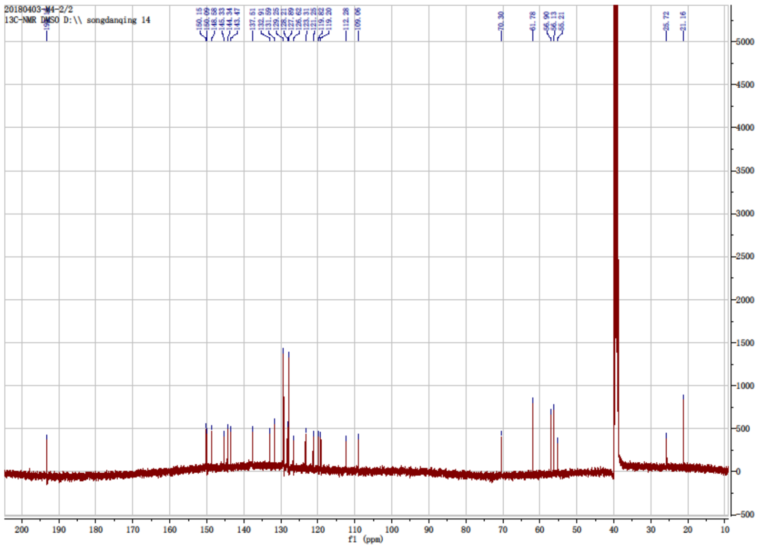
20180403-M4-2/2
13C-NMR DMSO D:\\ songdanqing 14
f1 (ppm)

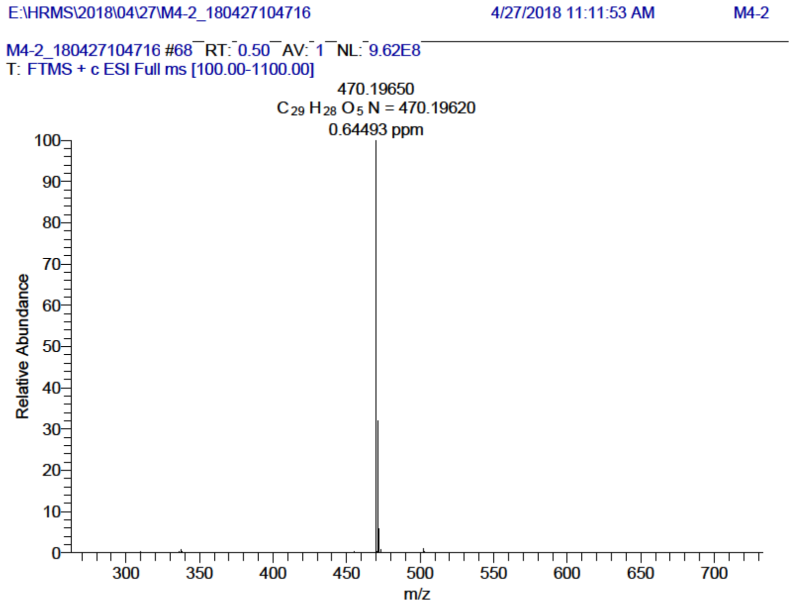
E:\HRMS\2018\04\27\M4-2_180427104716
4/27/2018 11:11:53 AM
M4-2
M4-2_180427104716 #68 RT: 0.50 AV: 1 NL: 9.62E8
T: FTMS + c ESI Full ms [100.00-1100.00]
470.19650
C 29 H 28 O 5 N = 470.19620
0.64493 ppm
Relative Abundance
m/z

5f

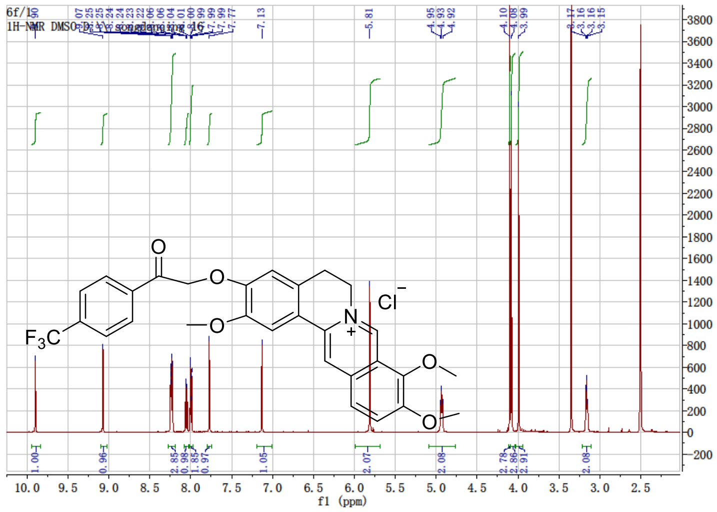
1H-NMR DMSO
Cl
F3C
f1 (ppm)

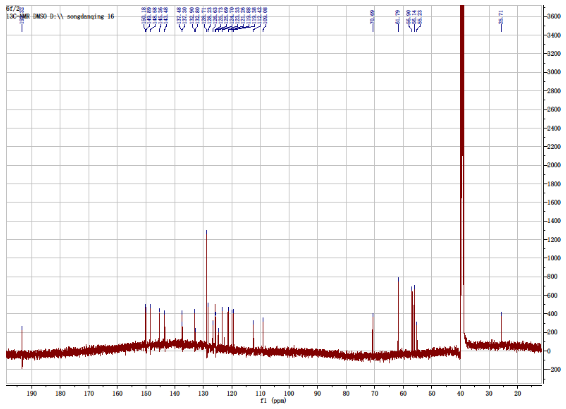

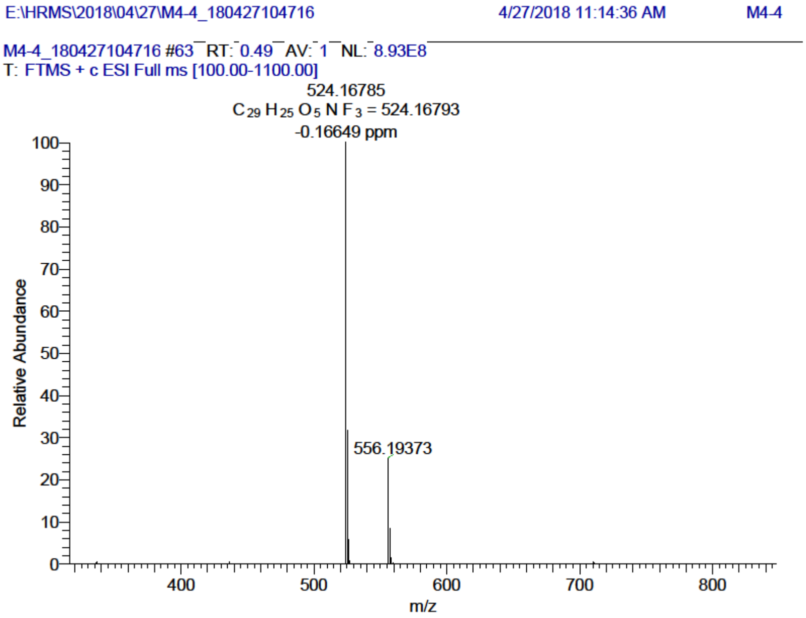
E:\HRMS\2018\04\27\M4-4_180427104716
4/27/2018 11:14:36 AM
M4-4
M4-4_180427104716 #63 RT: 0.49 AV: 1 NL: 8.93E8
T: FTMS + c ESI Full ms [100.00-1100.00]
524.16785
C29 H25 O5 N F3 = 524.16793
-0.16649 ppm
556.19373
Relative Abundance
m/z

5g

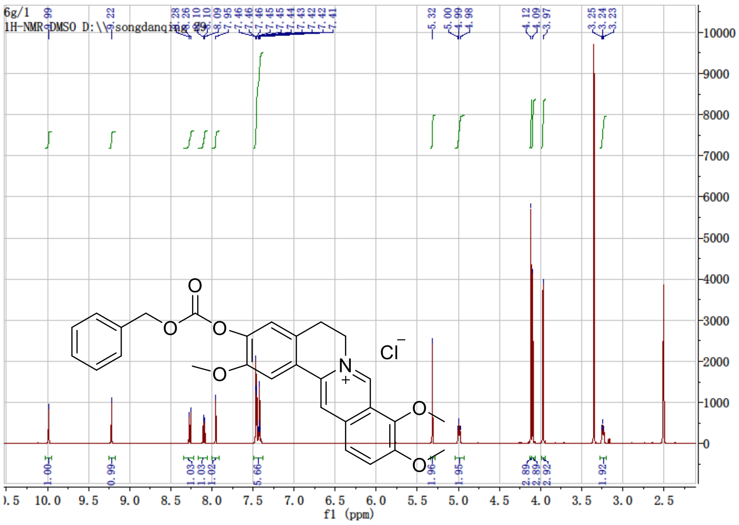
6g/1
1H-NMR-DMSO D:\\songdanqing 29
f1 (ppm)
Cl⁻

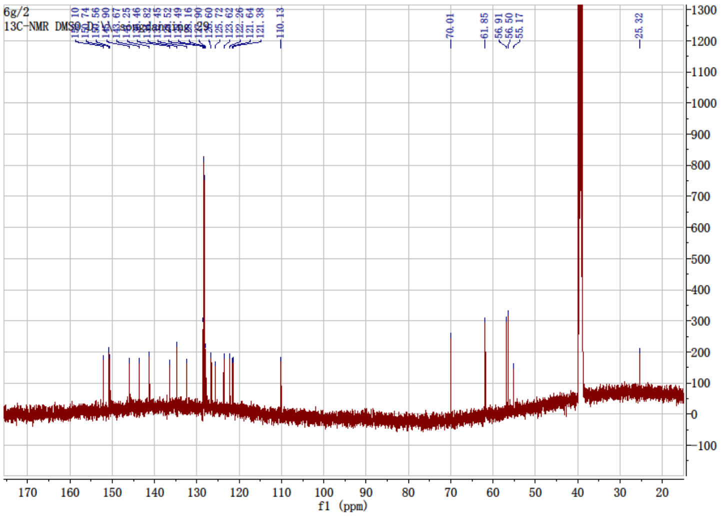
6g/2
13C-NMR DMSO D:\\songdanqing 29
f1 (ppm)

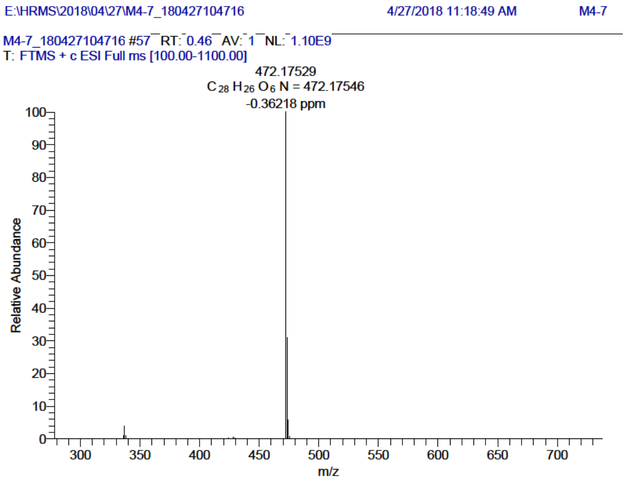
E:\HRMS\2018\04\27\M4-7_180427104716
4/27/2018 11:18:49 AM
M4-7
M4-7_180427104716 #57 RT: 0.46 AV: 1 NL: 1.10E9
T: FTMS + c ESI Full ms [100.00-1100.00]
472.17529
C 28 H 26 O 6 N = 472.17546
-0.36218 ppm
Relative Abundance
m/z
